# Supplementary material for: Counterion docking: a general approach to reducing energetic disorder in doped polymeric semiconductors
Source: Nat Commun. 2024 Jun 11;15:4972. doi: 10.1038/s41467-024-49208-x (PMC11166965; doi:10.1038/s41467-024-49208-x)
Supplement: Supplementary file 1 — Supplementary Information [file 41467_2024_49208_MOESM1_ESM.pdf]

Supplementary Information

for

Counterion docking: a general approach to reducing energetic disorder in doped polymeric semiconductors

*Miao Xiong[1,2#], Xin-Yu Deng[1#], Shuang-Yan Tian[1], Kai-Kai Liu[1], Yu-Hui Fang[2], Juan-Rong Wang[1], Yunfei Wang[3], Guangchao Liu[1], Jupeng Chen[1], Diego Rosas Villalva[4], Derya Baran[4], Xiaodan Gu[3], Ting Lei[1]**

[1]Key Laboratory of Polymer Chemistry and Physics of Ministry of Education, School of Materials Science and Engineering, Peking University, Beijing 100871, China.

[2]Beijing National Laboratory for Molecular Science, College of Chemistry and Molecular Engineering, Peking University, Beijing 100871, China.

[3]School of Polymer Science and Engineering, Center for Optoelectronic Materials and Devices, The University of Southern Mississippi, Hattiesburg, MS 39406, USA.

[4]Materials Science and Engineering Program (MSE), Physical Sciences and Engineering Division (PSE), King Abdullah University of Science and Technology (KAUST), Thuwal, 23955-6900, Saudi Arabia.

*Correspondence and requests for materials should be addressed to T.L. (tinglei@pku.edu.cn).

#These authors contributed equally to this work.

This file includes:

Section 1. Computer-aided counterion docking technique

Section 2. Molecular dynamic simulation and analysis

Section 3. Supplementary figures and tables

Section 1. Computer-aided counterion docking method

In this work, all density functional theory (DFT) calculations were performed using the Gaussian 16 package[1]. The counterion docking simulation was performed using a CDOCKER[2] protocol in the Discovery Studio software package. The counterion docking approach includes the following 3 steps:

1.1 Modeling the 3D stacking structure of the target polymer. Here, we extracted three π-stacked dimers from the MD supercell of the target polymers, P(PzDPP-2FT) and *o*FBDPPV (Fig. S1). The procedures for constructing the MD supercell of the two polymers have been described in previous works[3,4,5].

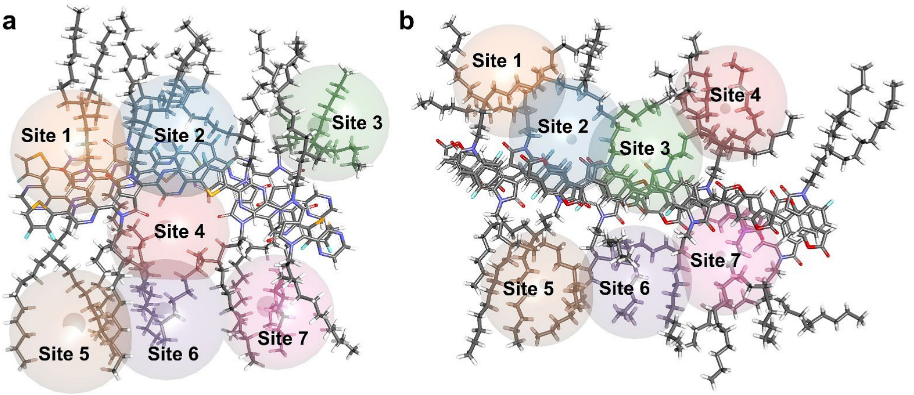

Figure S1. 3D π-stacked dimers of **a**, P(PzDPP-2FT) and **b**, *o*FBDPPV. The potential binding sites within the polymer structure are colored by different colored spheres.

1.2 Finding the most stable binding sites and fast counterion docking. Since dopants tend to insert into the sidechain regions with less disturbance to the molecular packing[4,6], we generated several potential binding sites within the polymer structure (Fig. S1). To identify the most stable binding sites, we performed docking with three representative cations: $BtMA^+$, $MPPyr^+$, and BPy^+. We then compared the interaction energies between the target polymers and the counterions (Fig. S2). Notably, for P(PzDPP-2FT), we observed that site 4, formed by the zigzag backbones and sidechains, exhibited significantly higher binding energy (above 20 kcal/mol) for all three cations compared to the other sites (less than 16 kcal/mol) (Fig. S2a). These findings suggested that the “cavity” formed by the zig-zag backbones could provide stronger stabilizing energy for the dopant counterions. On the contrary, *o*FBDPPV with linear backbones showed weaker binding energy to the same counterions (Fig. S2b). However, both polymers

displayed a similar trend of increasing affinities for all cations when the binding sites were closer to the backbones (such as site 1-4 for P(PzDPP-2FT) and site 1, 2&7 for *o*FBDPPV).

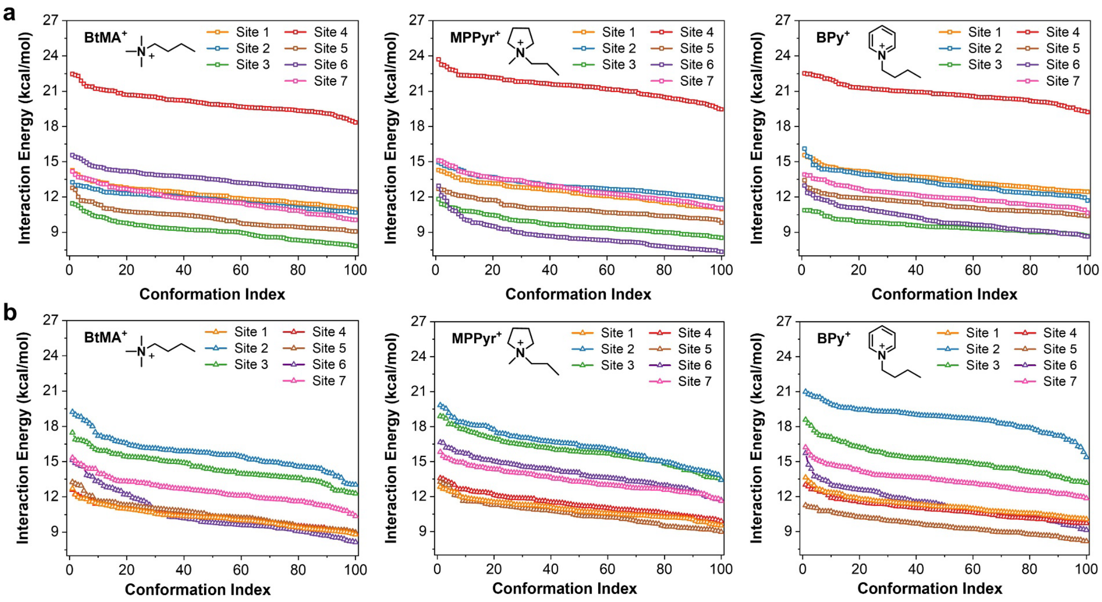

Figure S2. CDOCKER interaction energies between the counterions and the polymers: **a**, P(PzDPP-2FT) and **b**, *o*FBDPPV. For each counterion, 100 docking conformations were generated and analyzed.

A total of 28 common cations, classified into 9 classes (Fig. S3a), were docked into the final refined binding pocket. Specifically, site 4 was used for P(PzDPP-2FT), and site 2 was used for *o*FBDPPV. For each counterion, 100 docking poses were generated and the interaction energy was obtained by averaging the CDOCKER interaction energy from the top 10 most stable docking poses. As shown in Fig. S3b, there was a noticeable increase in the interaction energy between the counterions and P(PzDPP-2FT) as the length of the ion's alkyl chain increases (e.g., $BtMA^+$ *vs.* $MtBA^+$). Additionally, cations with aromatic structures exhibited stronger interactions compared to alkyl ions with similar sidechain lengths (e.g., BPy^+ *vs.* $BtMA^+$). Fig. S3c showed a similar trend for the interaction energy between counterions and *o*FBDPPV.

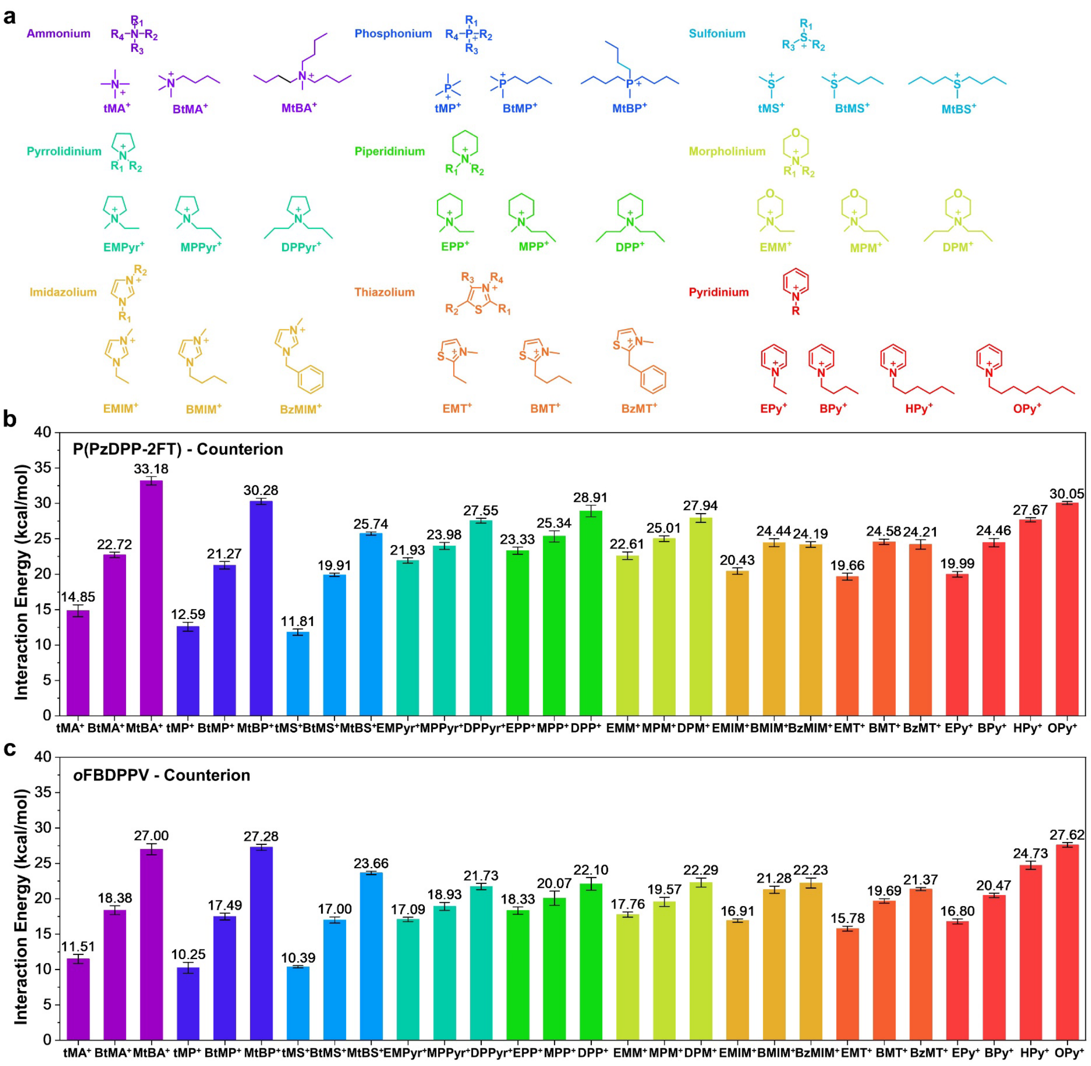

Figure S3. **a**, Chemical structures of 28 cations. Calculated averaged maximum interaction energies with the polymers: **b**, P(PzDPP-2FT) and **c**, *o*FBDPPV.

Table S1. Summary of the abbreviations and full names of the 28 counterions.

Abbreviation	Full name
**tMA**	Tetramethylammonium
**BtMA**	Butyltrimethylammonium
**MtBA**	Methyltributylammonium
**tMP**	Tetramethylphosphonium
**BtMP**	Butyltrimethylphosphonium
**MtBP**	Methyltributylphosphonium
**tMS**	Tetramethylsulfonium
**BtMS**	Butyltrimethylsulfonium
**MtBS**	Methyltributylsulfonium
**EMPyr**	1-methyl-1-ethylpyrrolidinium
**MPPyr**	1-methyl-1-propylpyrrolidinium
**DMPyr**	1-propyl-1-propylpyrrolidinium
**EPP**	1-methyl-1-ethylpiperidinium
**MPP**	1-methyl-1-propylpiperidinium
**DPP**	1-propyl-1-propylpiperidinium
**EMM**	1-methyl-1-ethylmorpholinium
**MPM**	1-methyl-1-propylmorpholinium
**DPM**	1-propyl-1-propylmorpholinium
**EMIM**	1-ethyl-3-methylimidazolium
**BMIM**	1-butyl-3-methylimidazolium
**BzMIM**	1-benzyl-3-methylimidazolium
**EMT**	1-ethyl-2-methylthiazolium
**BMT**	1-butyl-2-methylthiazolium
**BzMT**	1-benzyl-2-methylthiazolium
**EPy**	1-ethylpyridinium
**BPy**	1-butylpyridinium
**HPy**	1-hexylpyridinium
**OPy**	1-octylpyridinium

1.3 DFT calculations. The semiempirical docking approach neglects diatomic differential overlap approximation which is known to underestimate binding affinities significantly[7]. Thus, we supplemented DFT calculations on the most stable binding geometry obtained from docking. As illustrated in Fig. S4, the DFT-derived binding energy of different counterions and P(PzDPP-2FT) followed the same trend of CDOCER interaction energy in Fig. S3b, providing evidence for the credibility of the docking method.

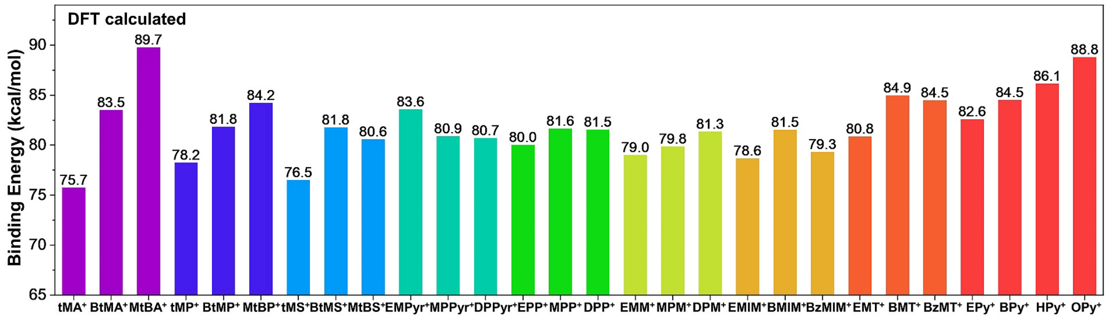

Figure S4. DFT-calculated binding energies of the most stable counterion-P(PzDPP-2FT) pairs obtained from CDOCKER. DFT calculation was performed at B3LYP/6-31+G(d,p) level with counterpoise correction.

Section 2. Molecular dynamic simulation and analysis.

2.1. Method for molecular dynamic simulation.

In this work, all molecular dynamics simulations were performed using Material Studio[8]. A force field derived from the Deriding force field, combined with quantum-chemical calculations, was employed. Specifically, the torsion potentials between adjacent conjugated units were re-parameterized against ωB97X-D/6-311G(d,p) calculation. For analysis, $MtBA^+$ was chosen as the representative alkyl cation, while HPy^+ and OPy^+ were selected to represent the aromatic cations. DFT-optimized geometries of the polymer tetramers and counterions were used as initial structures and atomic charges were resigned according to DFT-calculated RESP charges. Initially, the counterions were placed within the refined binding site selected by the counterion docking method (site 4 in Fig. S1a), where they exhibited the strongest NCIs with the polymers.

The microstructure of semicrystalline conjugated polymers is composed of three distinct regions: crystalline, disordered, and amorphous. Intrachain charge transport in amorphous regions is fast, and the primary limitation to charge transport is the intermolecular packing in the crystalline/disordered regions[9]. Therefore, the investigation of the counterion effects on the crystalline/disordered region is more critical than the amorphous region in charge transport. Given the difficulty of simulating counterion diffusion within the tightly packed crystalline regions over the timescales accessible to MD simulations, we focused our simulation on disordered regions, which provide more room for counterion diffusion. To explore potential packing modes of the polymer-counterion pairs, we created initial unit structures consisting of two polymer chains, each made of 4 monomers, along with one counterion for each chain. For the cell parameters, we set index a as the tetramer length and expanded π-π stacking distance c to 5 Å to simulate the disorder region. Meanwhile, we ensured that the lamella distance (index b) was large enough (~ 50 Å) to accommodate the alky sidechains. By performing multiple geometry optimizations, we gradually decreased the b value to around 28 Å and observed the reduction of the c value from 5 to 4 Å. This allowed us to obtain structures with appropriately relaxed

alky sidechains (Fig. S5).

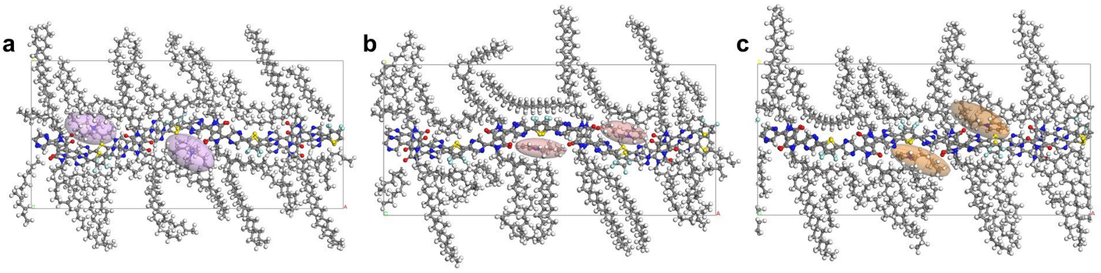

Figure S5. The unit cell of P(PzDPP-2FT) with **a,** $MtBA^+$, **b,** HPy^+, and **c,** OPy^+, which were rendered as purple, red, and orange transparent ellipsoids, respectively.

To search for the most stable molecular structures, we followed a 4-step thermodynamic approach according to the previous simulation for the disorder region of the polymers: (i) Unit cells containing two polymer chains and two counterions were built under periodic boundary conditions to simulate an infinite system. (ii) Both polymer-counterion systems underwent optimization and were quenched at the molecular mechanics level (NPT, T = 300 K, quench frequency = 1 ps, simulation time = 100 ps) until the energy between two consecutive structures no longer decreased; (iii) The quenched dynamics were continued for an additional 100 ps at higher temperatures (600 K and 1000 K) to explore more stable structures; (iv) The molecular structures obtained at step iii were further quenched with a longer simulation time (500 ps) at increasing temperatures (300 K, 600 K, and 1000 K).

Based on the stable unit structures, supercells made of 3 layers of 8 π-stacked dodecamers were constructed. All supercells underwent a series of consecutive molecular dynamics: (i) To facilitate counterion diffusion, the entire systems were submitted to a high-temperature dynamics simulation (NPT, P = 1 atm, T = 500 K, 50 ps), providing a full relaxation along and between the molecules; (ii) Supercells were then thermalized and relaxed with two consecutive 200 ps-long dynamics calculation (NPT, P = 1 atm, T = 298 K, snapshots saved every 1 ps); (iii) The dynamics with the same condition were continued for an additional 200 ps to collect data. The snapshots from these dynamic simulations are presented in Fig. S6. As anticipated from the counterion docking, the alkyl counterion $MtBA^+$ gradually diffused into the sidechain packing regions, moving away from the polymer backbones (Fig. S6a). In contrast,

most of the HPy^+ and OPy^+ ions remained close to the polymer backbones (Fig. S6b-c).

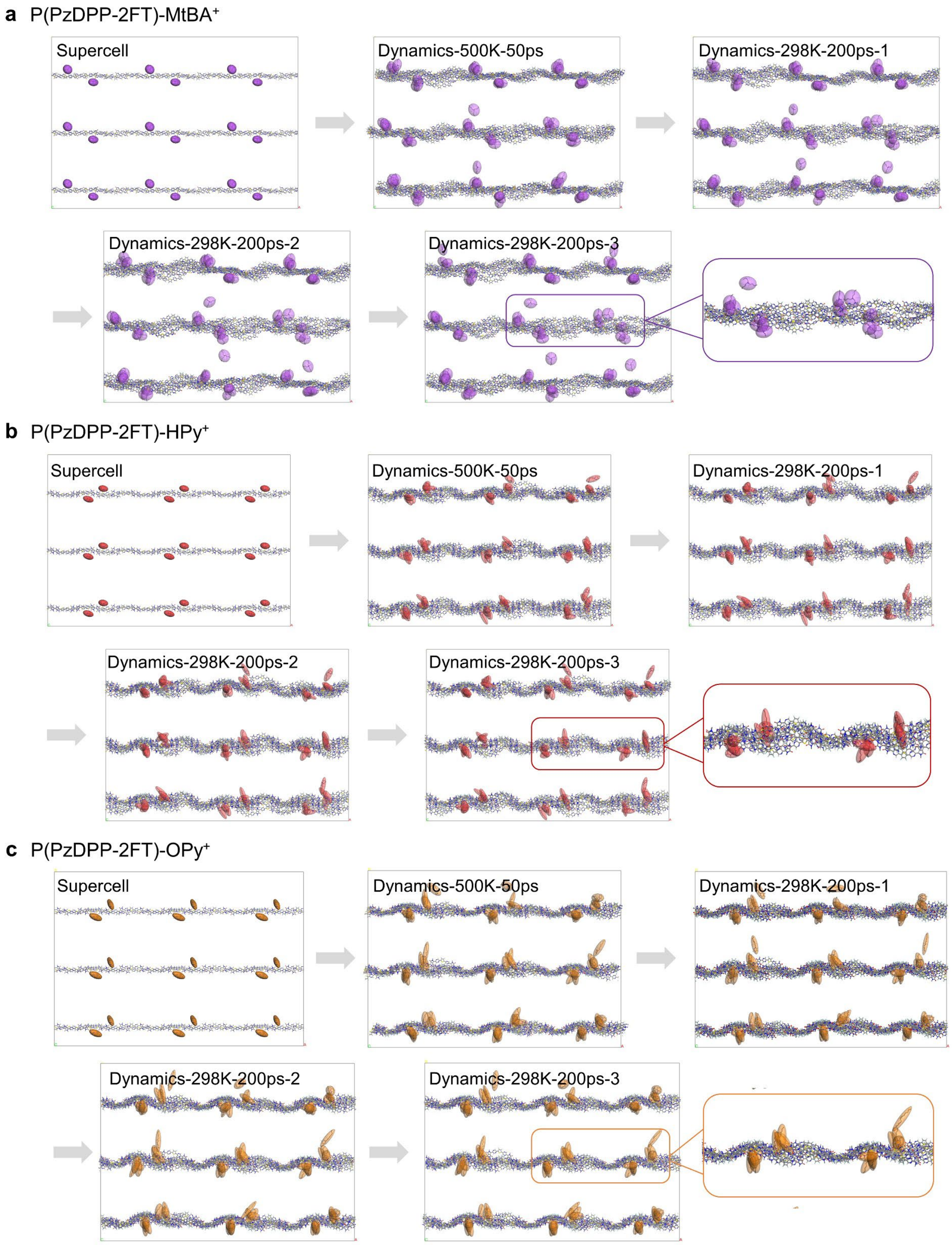

Figure S6. The dynamic diffusion process of **a,** $MtBA^+$, **b,** HPy^+, and **c,** OPy^+ in P(PzDPP-2FT) supercell. To enhance clarity, the alkyl sidechains of the polymer were hidden.

2.2. Analysis for counterion distribution.

The initial position for both $MtBA^+$ and HPy^+ was almost identical, as shown in Fig. S7a-b. After dynamic diffusions, statistical analysis showed that $MtBA^+$ cations were located further away from the polymer backbone compared to HPy^+ and exhibited a broader distribution ranging from 4 to 19 Å (Fig. S7c). In addition, we also employed statistical analysis for ion-ion distance (R) distribution (Fig. S7d). The enhanced stabilization of HPy^+ around the polymer backbone results in a smaller average ion-ion distance (R) of 8.26 Å compared to $MtBA^+$ (8.96 Å).

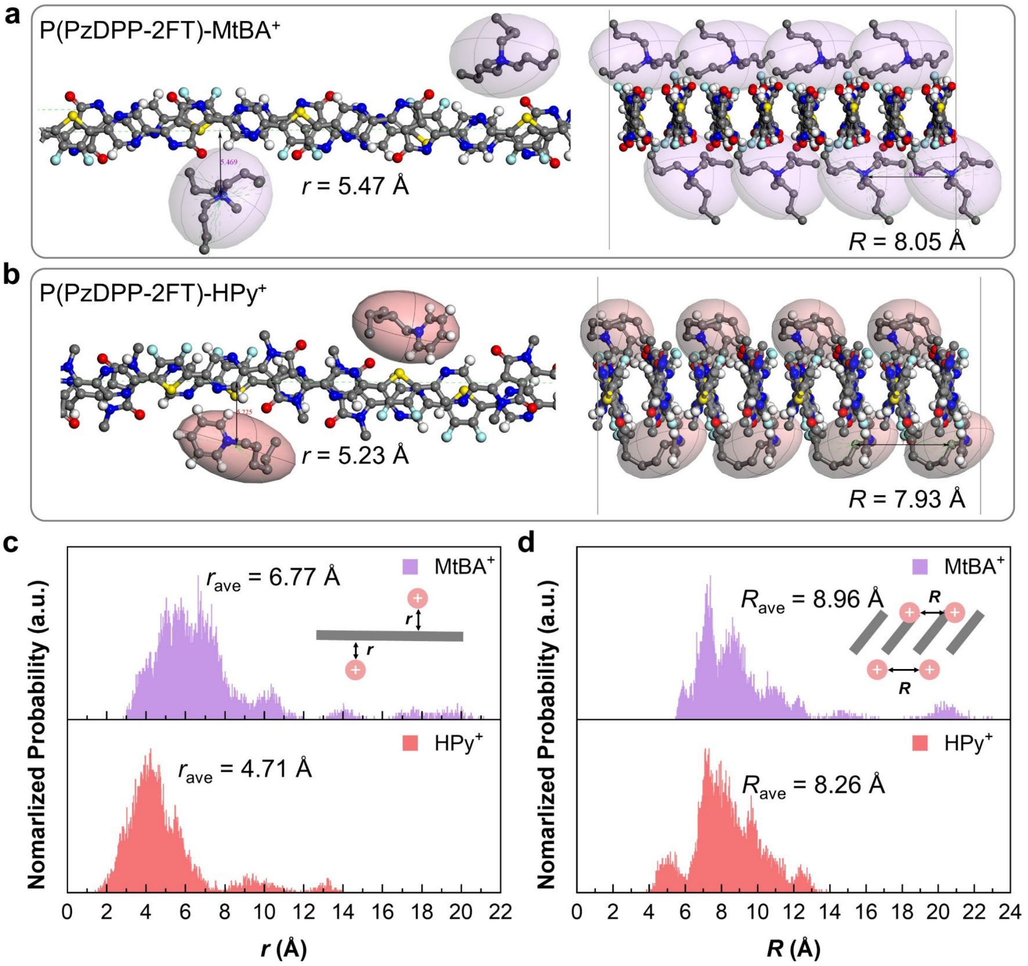

Figure S7. Front (left) and side (right) views of the initial positions of counterions before dynamic simulations for **a,** $MtBA^+$ and **b,** HPy^+. **c**, Statistical analysis of the polymer-counterion distance (r), defined as the distance between the geometric center of the adjacent polymer backbone and the geometry center of a counterion. **d,** Statistical analysis of the counterion-counterion distance (R), representing the distance between the geometry centers of counterions. All statistical analyses are obtained from the last 200 ps of dynamic simulations.

To further facilitate the diffusion of counterions, a series of high-temperature MD simulations were conducted. The process began with a constant temperature and pressure (NPT) simulation, where the system was maintained at 1000 K under a pressure of 1 atmosphere for 200 ps. Following this, two additional 200-ps dynamic simulations were executed at sequentially reduced temperatures of 600 K and 300 K (Fig. S8 and Fig. S9). As the temperature increased to 1000 K, the polymer backbone, which initially had a zig-zag backbone conformation, straightened to a near-linear structure (Fig. S8b and Fig. S9b). Concurrently, the distribution of counterions became broader (Fig. S10 and S11), indicating that the molecules within the systems were fully relaxed. Throughout the heating-to-cooling process, HPy^+ was observed to consistently locate near the polymer backbone, while $MtBA^+$ exhibited a more chaotic distribution (Fig. S8 and Fig. S9). Statistical analysis further confirmed that the polymer-counterion distribution of HPy^+ kept almost unchanged throughout the process, whereas the distribution for $MtBA^+$ broadened significantly (Fig. S10). This chaotic distribution of $MtBA^+$ within the polymer led to a more dispersed and multimodal counterion-counterion distance distribution, with the average distance R_{avg} increasing markedly from 8.96 to 13.50 Å (Fig. S11). In contrast, the counterion-counterion distribution of HPy^+ remained consistent after this process, with only a slight expansion in the average distance R_{avg} to 10.36 Å. These results suggest that the stronger electrostatic interactions effectively stabilize HPy^+ around the polymer backbone, leading to a more ordered distribution. This stable positioning may also contribute to a more pronounced overlap of Coulombic traps, which could influence the overall electrostatic landscape within the polymer system. The high-temperature simulations accelerate the counterion diffusion and demonstrate the difference between $MtBA^+$ and HPy^+.

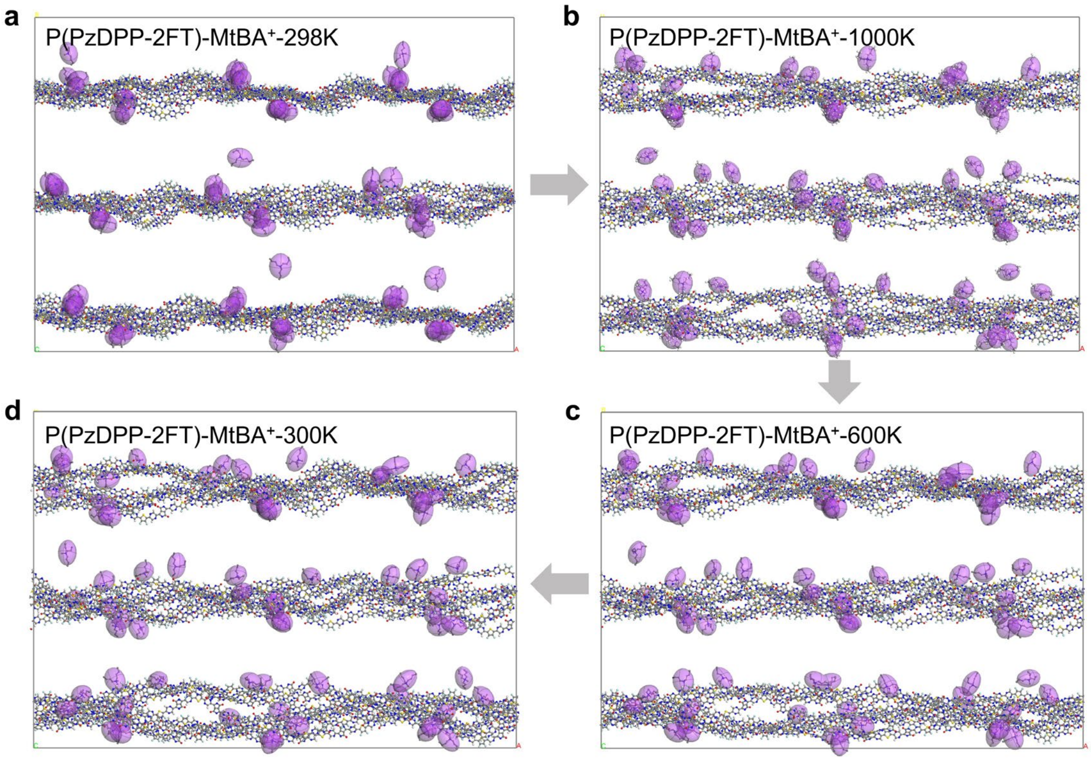

Figure S8. MD simulations of $MtBA^+$ in P(PzDPP-2FT) supercell for 200 ps at different temperatures: **a,** 298 K, **b,** 1000 K, **c,** 600 K, and **d,** 300 K (annealed).

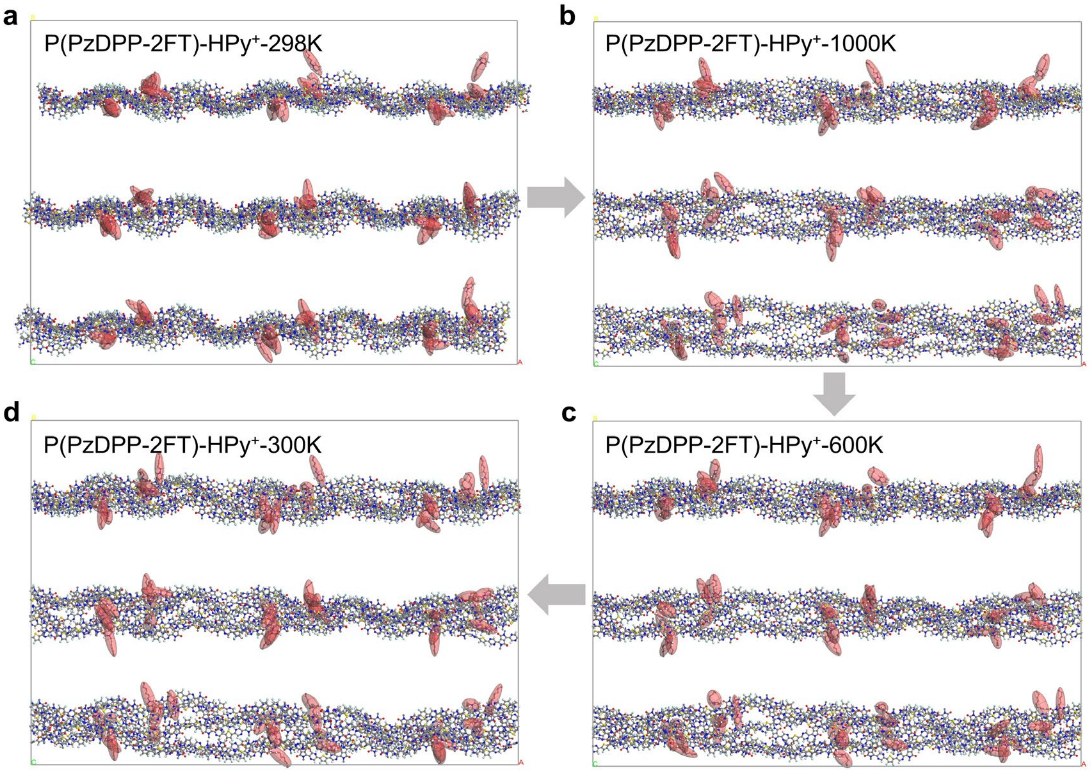

Figure S9. MD simulations of HPy^+ in P(PzDPP-2FT) supercell for 200 ps at different temperatures: **a,** 298 K, **b,** 1000 K, **c,** 600 K, and **d,** 300 K (annealed).

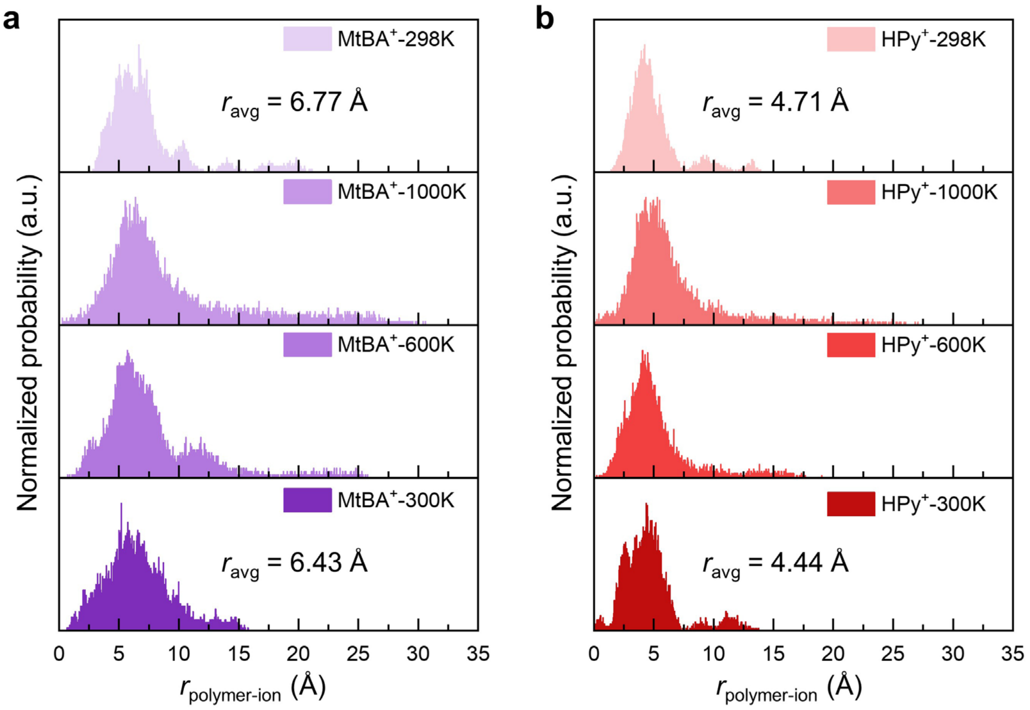

Figure S10. Statistical analysis of the polymer-counterion distance r of **a,** $MtBA^+$ and **b,** HPy^+. The distance r is defined as the distance between the geometry center of a counterion and the geometric center of the nearest polymer backbone.

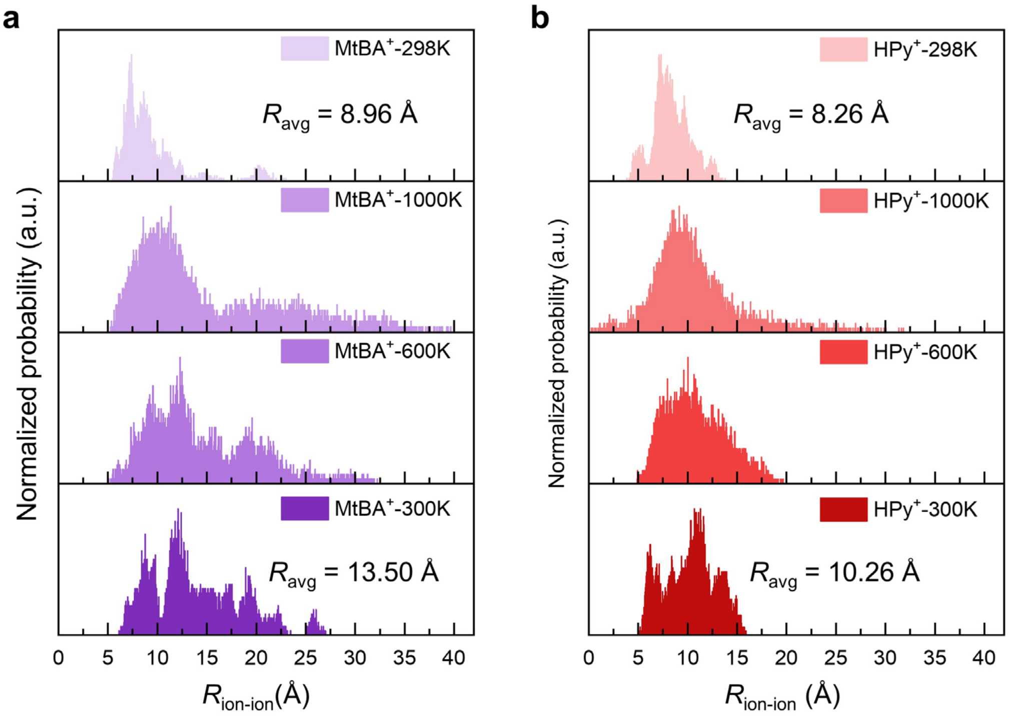

Figure S11. Statistical analysis of the counterion-counterion distance R of **a,** $MtBA^+$ and **b,** HPy^+. The distance R is defined as the distance between the geometry centers of the nearest counterions.

2.3. Energy decomposition analysis.

Employing energy decomposition analysis (EDA) based on the force field, we identified two major ingredients of NCIs: electrostatic (ELS) and van der Waals (vdW) interactions. The ELS potential is computed from the partial atomic charges[10] and the vdW interactions include repulsive interaction due to the Pauli repulsion effect (E_{rep}) and attractive dispersion interaction (E_{disp})[11]. The introduction of counterions notably enhances NCIs, suggesting unignorable NCIs between the polymer and counterions (Fig. S12a). Specifically, the ELS interactions in polymer with HPy^+ and OPy^+ notably exceed those in polymer-$MtBA^+$. In contrast, polymer-$MtBA^+$ exhibits more substantial vdW interactions than both polymer-HPy^+ and polymer-OPy^+. As shown in Fig. S12b, within polymer-$MtBA^+$, ELS interactions account for 64.8% of the total NCIs, with vdW interactions contributing the remaining 35.2%. However, in the polymer-HPy^+ or OPy^+ systems, the composition of NCIs leans more towards ELS interactions, which constitute approximately 80% with vdW interactions contributing about 20%. To better understand these differences, we evaluated the interaction energies between the counterions and separate parts of the polymer (backbone and sidechain). The ELS interactions significantly influence the attraction between the anionic polymer backbone and cationic counterions (Fig. S12c), and contribute to the repulsion between the polymer sidechain and counterions (Fig. S12d). Additionally, vdW interactions are key in facilitating attractive interactions between the polymer sidechain and counterions. Particularly for $MtBA^+$, the attractive vdW interactions outweigh the repulsive ELS interactions with the polymer sidechain, leading to more pronounced attractive interactions with the polymer sidechain compared to the other two aromatic cations.

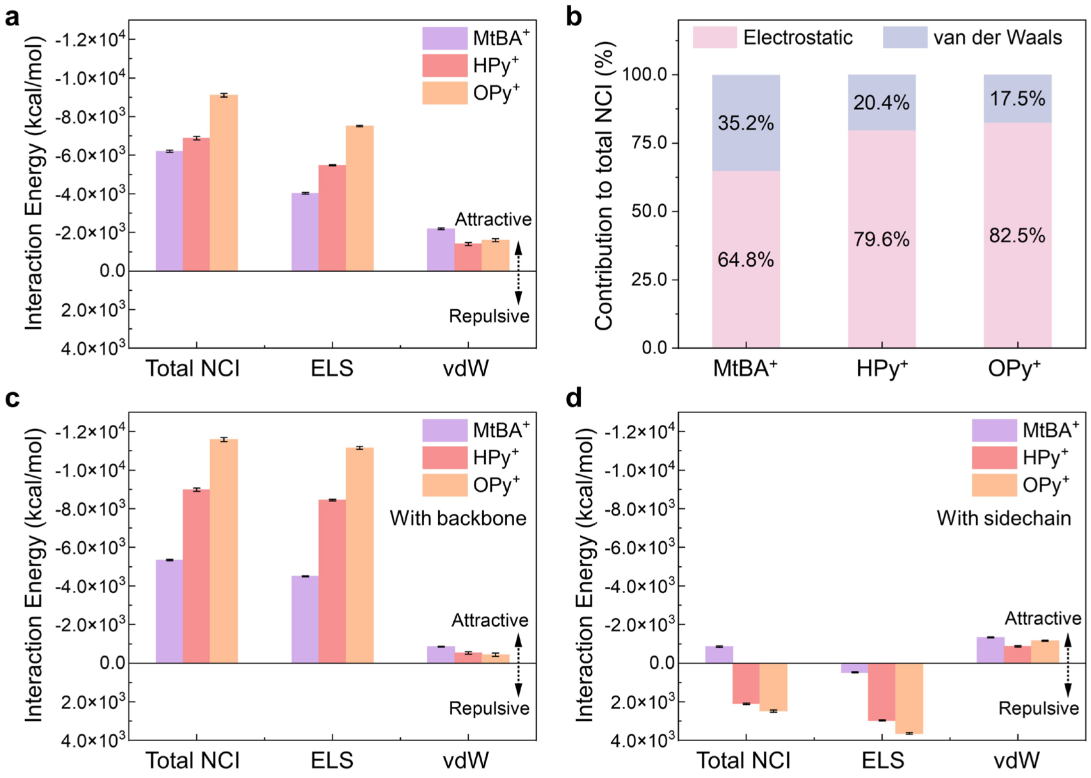

Figure S12. a, Energy decomposition analysis (EDA) based on the force field for the interaction between 24 P(PzDPP-2FT) polymer chains and 72 counterions within the supercell. **b**, Quantitative analysis depicting the contribution of electrostatic or van der Waals interaction to the overall NCIs between P(PzDPP-2FT) and counterions. **c,** EDA for the interactions between the polymer backbone and counterions. **d,** EDA for the interactions between the polymer sidechain and counterions. Error bars of interaction energies represent the variation during the last 200 ps dynamic simulations.

To better understand ELS and vdW interactions, we also performed EDA based on DFT-calculated wavefunctions using a recently reported sobEDAw method[12]. sobEDAw decomposes intermolecular interaction energy as follows:

$$\Delta E_{int} = \Delta E_{els} + \Delta E_{xrep} + \Delta E_{orb} + \Delta E_{disp}$$

where ΔE_{orb} and ΔE_{xrep} represent orbital and exchange-repulsion terms, respectively; ΔE_{els} and ΔE_{disp} denote ELS and dispersion interactions, respectively. The interaction energies between counterions and different parts of the polymer (backbone and sidechain) were summarized in Table S2. HPy^{+} and OPy^{+} consistently exhibit stronger ΔE_{els} with the polymer backbone (−56.0 kcal/mol and −59.3 kcal/mol, respectively) compared to $MtBA^{+}$ (−52.8 kcal/mol). Conversely, $MtBA^{+}$ displays a stronger dispersion interaction with the sidechain (−17.0 kcal/mol) compared to HPy^{+} and OPy^{+}

(−8.29 kcal/mol and −11.8 kcal/mol, respectively). EDA analyses based on both force field and wavefunctions revealed that significant electrostatic interactions effectively stabilize counterions around the polymer backbone (as observed with HPy^+ and OPy^+). Although vdW interactions contribute less to overall NCIs compared to electrostatic energy, their accumulation across multiple polymer sidechains remains crucial for counterion diffusion and stabilization.

Table S2. Components of the interaction energies (kcal/mol) derived by sobEDAw method.

Polymer	Counterion	ΔE_{int}	ΔE_{els}	ΔE_{orb}	ΔE_{rexc}	ΔE_{disp}
**Backbone**	**$MtBA^+$**	−57.4	−52.8	−11.6	25.7	−8.71
	**HPy^+**	−68.2	−56.0	−13.0	15.3	−14.4
	**OPy^+**	−65.3	−59.3	−14.2	22.9	−14.6
**Sidechain**	**$MtBA^+$**	−13.1	−3.15	−5.91	13.0	−17.0
	**HPy^+**	−7.00	−1.28	−2.67	5.25	−8.29
	**OPy^+**	−8.65	−2.14	−4.16	9.48	−11.8

2.4. Counterion effect on polymer conformation.

The transition from a neutral state to a charged state indeed enhances the polymer backbone planarity, as demonstrated by a previous study[13]. We compared the backbone planarity of the P(PzDPP-2FT) dimer in its neutral and doped states (Fig. S13). In the charged state, the length of the C-C linkage bond between repeating units shortens from 1.45 Å to 1.43 Å, indicating a more quinoid structure. Furthermore, the torsion barrier height between repeating units increases from 8.68 to 13.3 kcal/mol. Besides, we noticed that interactions between the polymer backbone and the dopant counterion contribute to further planarization of the doped conjugated backbone. It has been well-studied that intramolecular noncovalent interaction can “lock” the backbone conformation[14,15]. Notably, NCIs also exist between the polymer and the dopant counterion (Fig. S14). We observed that, despite a slight increase in the C-C linkage bond length of $(PzDPP)_2^{1-}$-HPy^+ (or $MtBA^+$) compared to $(PzDPP)_2^{1-}$, the torsion barrier height significantly increased to around 19 kcal/mol in the presence of counterions (Fig. S13). This result indicates that intermolecular NCIs also contribute to enhancing the backbone planarity, which we refer to as “counterion locking”.

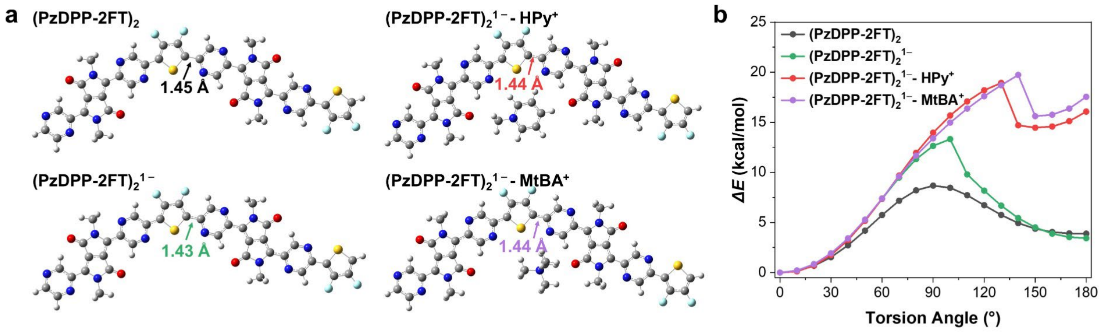

Figure S13. a, DFT-optimized structure of the P(PzDPP-2FT) dimer in neutral and doped states. **b,** Relaxed potential energy scan of the torsion angle between the repeating units of P(PzDPP-2FT) dimer in neutral and doped states.

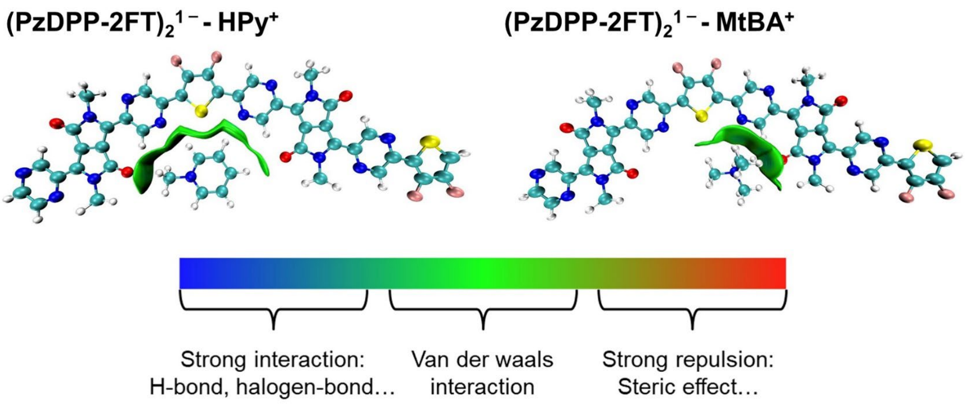

Figure S14. Visualization of the intermolecular interaction between the polymer backbone and counterions using the independent gradient model (IGM) analysis[16].

To assess the "counterion locking" effect, we use the same force field for pristine polymer and polymer with counterions in molecular dynamic simulations. As shown in Fig. S15, the polymer with $MtBA^+$ counterions exhibited broadened torsion angle distributions, especially for the DPP-Pz torsion angle directly linked to alkyl sidechains. However, the polymer with HPy^+ exhibited an even narrower torsion angle distribution compared to the pristine polymer, indicating enhanced backbone planarity. These results suggest that when the counterions are located around the polymer backbones, they can effectively "lock" the polymer backbone rotations through NCIs.

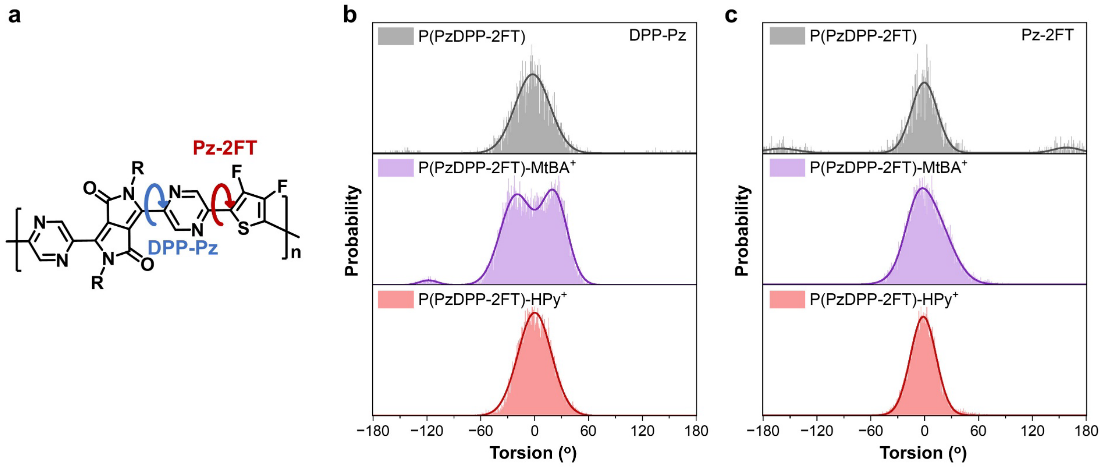

Figure S15. a, Chemical structure of P(PzDPP-2FT). The torsion-angle DPP-Pz and Pz-2FT were indicated by blue and red arrows, respectively. Torsion-angle distributions of **b**, DPP-Pz, and **c**, Pz-2FT segments in the pristine P(PzDPP-2FT) and the polymer with $MtBA^+$ or HPy^+.

We also observed a counterion effect on the polymer packing. As shown in Fig. S16, the diffusion of $MtBA^+$ into the sidechain regions led to increased sidechain disorder (lamellar expansion) and a greater twisting of the polymer backbone (larger π-π distance). Conversely, HPy^+ near the polymer backbone contributed to a smaller lamellar distance, and the stronger NCIs between the charged polymer backbone and HPy^+ contributed to the suppression of backbone torsion and smaller π-π stacking distance. As a result, both the lamellar and π-π distances in polymer-HPy^+ end up being smaller than those in polymer-$MtBA^+$.

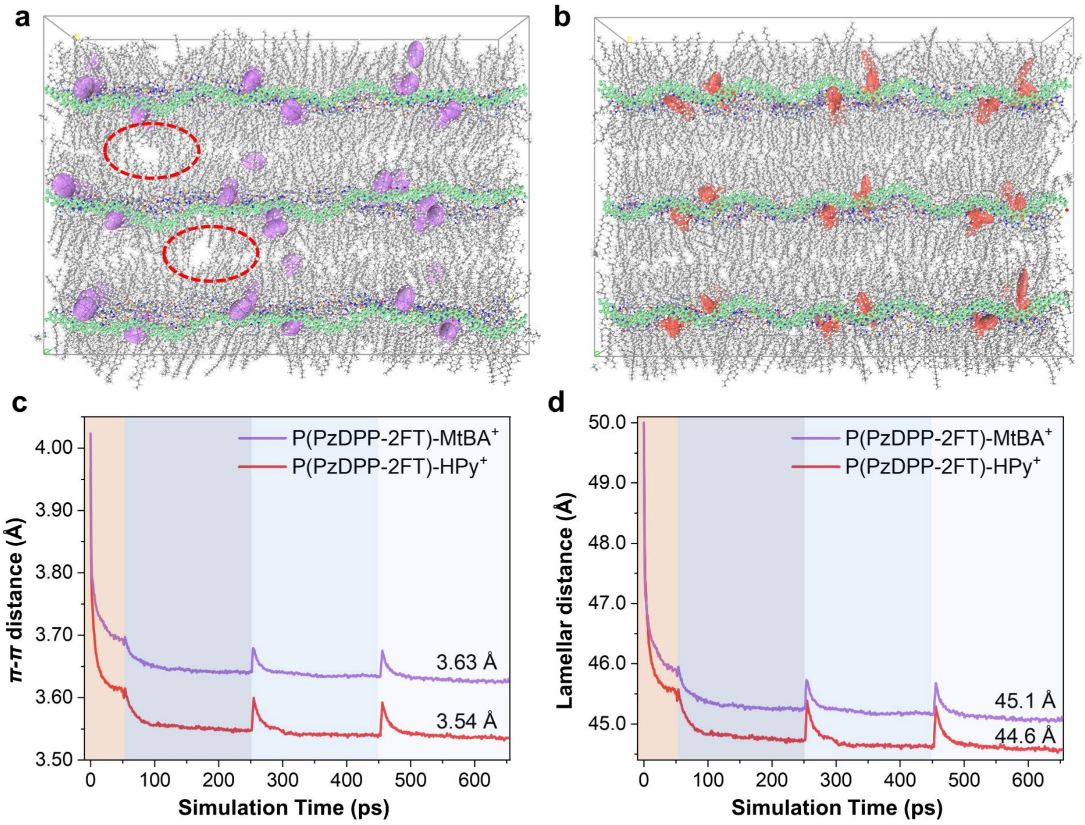

Figure. S16. MD simulation snapshot of the polymer P(PzDPP-2FT) with **a,** $MtBA^+$ or **b,** HPy^+ as the counterions. The disorder sidechain regions in P(PzDPP-2FT)-$MtBA^+$ are highlighted with red circles. Static analysis of **c,** lamellar distances, and **d,** π-π stacking distances for the supercell of the polymer with HPy^+ and $MtBA^+$.

To investigate the impact of counterions on the polymer electronic structure, we calculated the bulk orbital localization length (*LL*). The localization length for molecular orbitals of all the extracted polymer chains was considered[17,18]. The distribution of states near the band edge strongly correlates with the energetic disorder

and mobility of polymers[19,20]. Hence, we compared the localization length at the band edge (Table S3). The polymer with HPy^{+} exhibited an extended localization length at the band edge, approximately 40 Å, significantly larger than that of the undoped polymer (35 Å) and the polymer with $MtBA^{+}$ (32 Å). These findings indicate that appropriate counterions, such as HPy^{+}, could reduce the disorder in polymer backbone rotation and facilitate charge delocalization.

Table S3. Comparison of *LL* valuers of the original and ion-exchanged polymers.

	P(PzDPP-2FT)	P(PzDPP-2FT)-$MtBA^{+}$	P(PzDPP-2FT)-HPy^{+}
LL (E_{EDGE}) (Å)	35	32	40

2.5. Counterion effect on Coulomb potential landscape.

To explore the influence of the counterions on the Coulomb traps, we extracted the polymer-counterion pairs from the final molecular dynamics simulation and calculated the ELS energy V_C using a recently reported model[21]. This model describes the ELS interactions of the dopant counterion by considering the monopole (charge q) and quadrupole moment (Q). The formula to compute these interactions estimation reads:

$$V_C = \sum_i \left(\frac{-eq_i^p}{4\pi\epsilon_0\epsilon_r r_{ij}} + \frac{q_i^p}{8\pi\epsilon_0\epsilon_r} \frac{\boldsymbol{r}_i \boldsymbol{Q} \boldsymbol{r}_i}{r_i^5} \right)$$

where the dipole moments of all their studied dopant molecules are negligible. However, the counterions in our system can have substantial dipole moments, up to 14.9 Debye, and vary for different counterions, which cannot be disregarded (Fig. S17). Additionally, the initial formula simplifies the dopant counterion as a point charge. However, we observed that the electron density derived from the wavefunction exhibits a strong correlation with the molecular conformation, resulting in the quadrupole moment fluctuating with changes in ion conformations (Fig. S18). Considering the significant differences in charge distribution between alkyl counterion and aromatic counterions, treating them as point charges at the geometric center is inadequate. Therefore, we reevaluated the V_C, considering the partial charge and dipole moments of counterions. The revised formula we used is:

$$V_C = \sum_i \left(\sum_j \frac{q_j^c q_i^p}{4\pi\epsilon_0\epsilon_r r_{ij}} + \frac{q_i^p}{4\pi\epsilon_0\epsilon_r} \frac{\boldsymbol{D}\boldsymbol{r}_i}{r_i^3} + \frac{q_i^p}{8\pi\epsilon_0\epsilon_r} \frac{\boldsymbol{r}_i \boldsymbol{Q} \boldsymbol{r}_i}{r_i^5} \right)$$

Here, $\epsilon = \epsilon_0\epsilon_r$ is dielectric permittivity. i and j indexes the atoms within the polymer and counterions, respectively. q_i^p and q_j^c denote the partial atomic charges of the polymer and the counterions. The distance between atom i in polymer and atom j in counterion is represented by r_{ij}. $\boldsymbol{r}_i$ is a vector connecting polymer partial charges and counterion center-of-geometries; D and Q represent the counterion dipole moment and quadrupole moment, respectively.

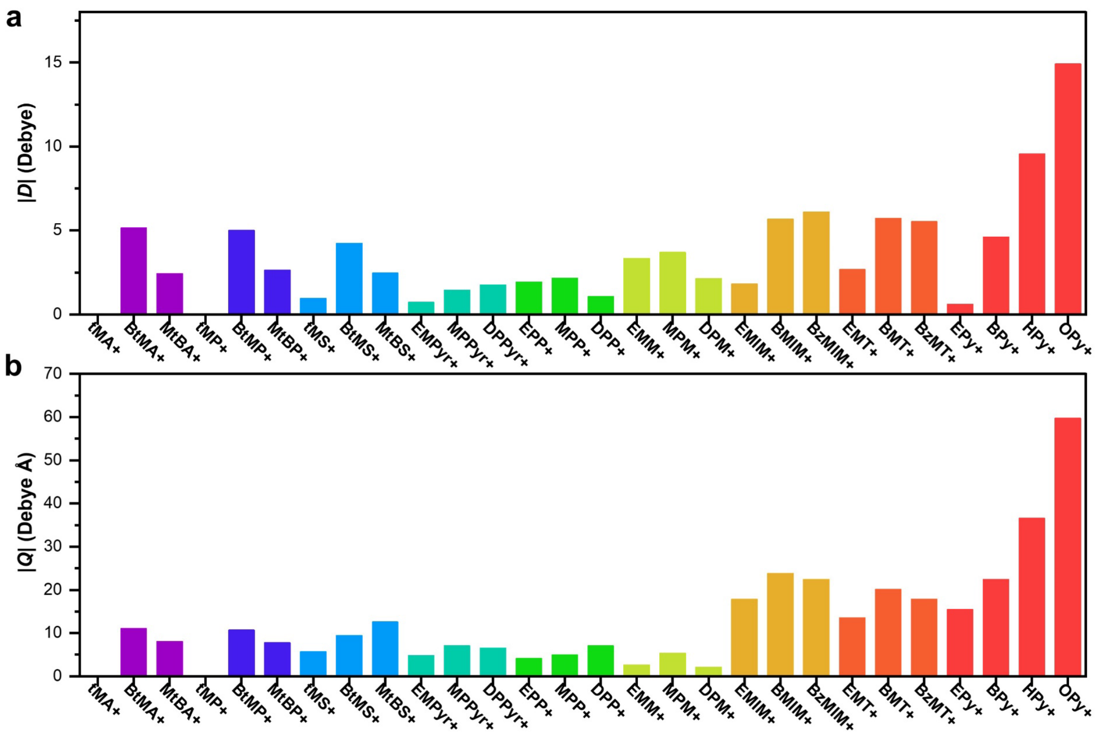

Figure S17. The magnitude of **a,** dipole moments and **b,** quadrupole moments calculated via DFT-optimized conformation for 28 cations.

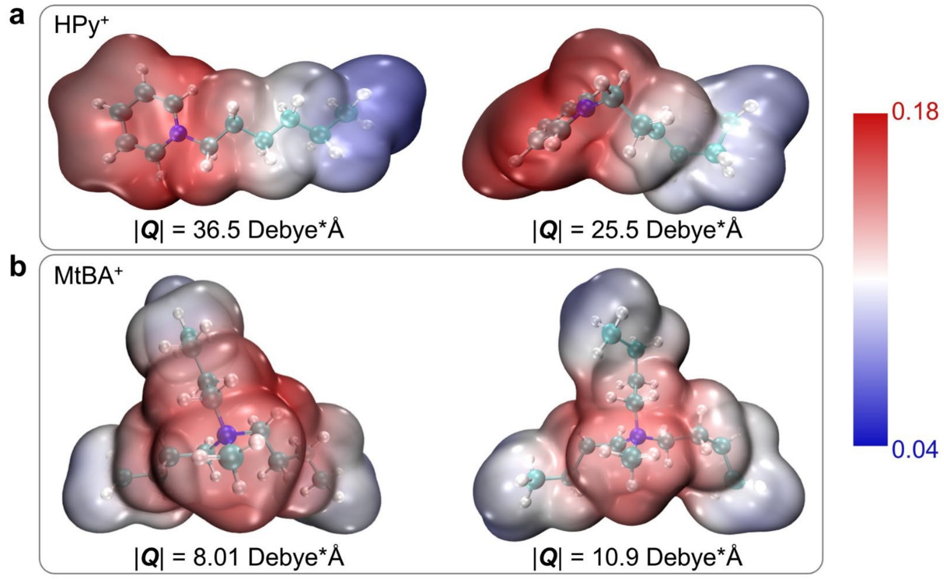

Figure S18. Electrostatic potential (ESP) mapped molecular vdW surfaces for **a,** HPy^+ and **b,** $MtBA^+$. The color scale represents ESP values ranging from 0.18 and 0.04 a.u. The corresponding conformation for each counterion is labeled with its quadrupole moment magnitude.

In the multipole expansion of V_C, the monopole term (charge-charge interaction) is the most significant contributor (Fig. S19). When the distance between the polymer

and counterion (r) decreases to below 5.0 Å ($1/r > 0.2$ Å^{-1}), the dipole and quadrupole interactions gradually increase (Fig. S19c-d). However, the impact of these short-range interactions remains quite modest. For HPy^+, the average dipole and quadrupole interactions are 0.038 eV and 0.012 eV, respectively. These values are considerably smaller than the average charge-charge interaction of −0.64 eV Å (Fig. S19b). $MtBA^+$ has smaller dipole and quadrupole moments (Fig. S17 and S18), resulting in average dipole and quadrupole interaction of around 0.016 eV and 0.0039 eV. It is important to note that the dipole interactions are an order of magnitude larger than the quadrupole term for both counterions. As a result, these short-range multipole interactions result in an up-shift in the electrostatic potential of 0.050 eV for HPy^+ and 0.016 eV for $MtBA^+$ (Fig. S19a). Nonetheless, these shifts are tiny when compared to the charge-charge interaction, and they barely change the electrostatic potential for both cations. Furthermore, a flat electrostatic potential at short distances has been observed, deviating from the classical Coulomb formula ($V_C \propto r^{-1}$). This effect was previously attributed to the quadrupole-charge interaction[21]. However, our analysis demonstrated that the charge distribution of the counterions also led to this deviation. The dipole and quadrupole components highlighted the importance of charge distribution on counterions[21,22]. We have tried to establish a connection between the dipole or quadrupole moments of the counterions and electrical conductivity (Fig. S20). It appears that polymers doped with counterions that possess substantial dipole and quadrupole moments can achieve higher electrical conductivity. However, a definitive quantitative relationship remains elusive. Therefore, we have redefined V_C to include the contributions from monopole, dipole, and quadrupole interactions, thereby providing a comprehensive assessment of their cumulative effects on the system.

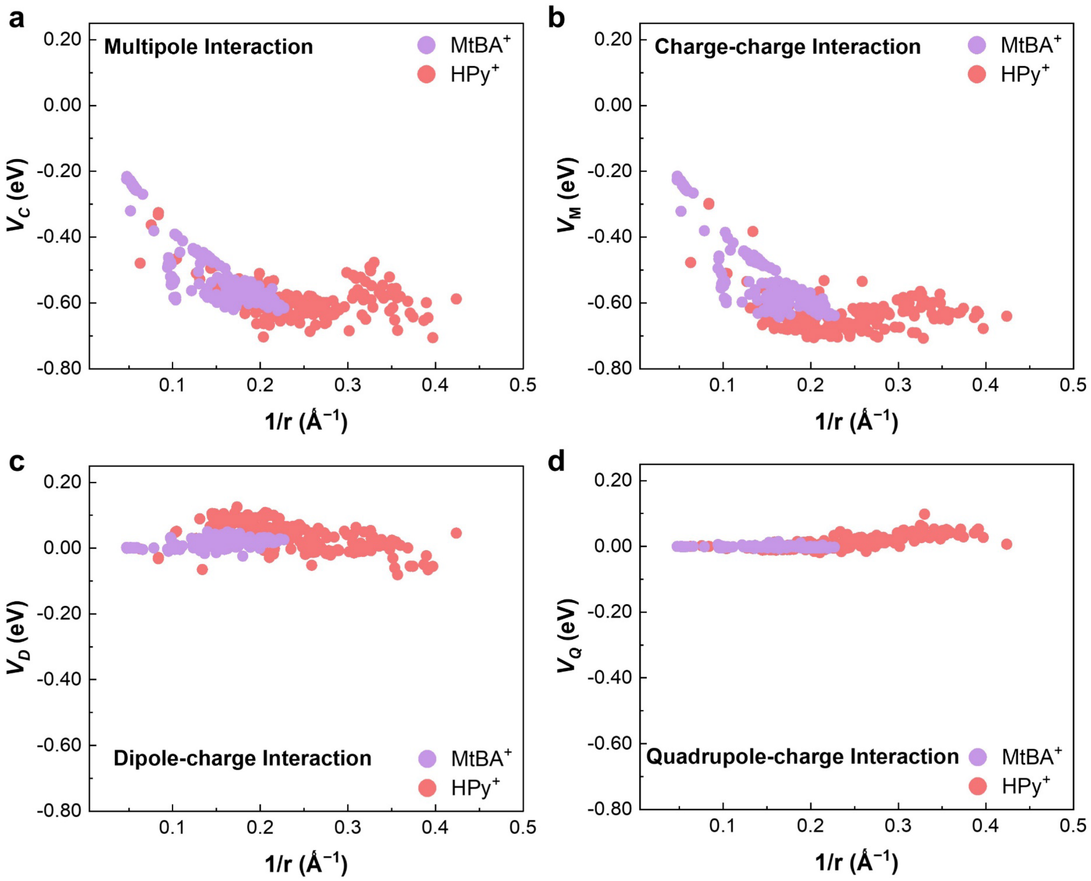

Figure S19. a, The distance-dependent V_C for P(PzDPP-2FT) with $MtBA^+$ and HPy^+. **b,** the monopole term (charge-charge interaction), **c,** the dipole term (dipole-charge interaction), and **d,** the quadrupole term (quadrupole-charge interaction) in the multipole expansion of V_C. The polymer-counterion distance (r) was measured between the geometry center of a counterion and the geometric center of the adjacent polymer backbone.

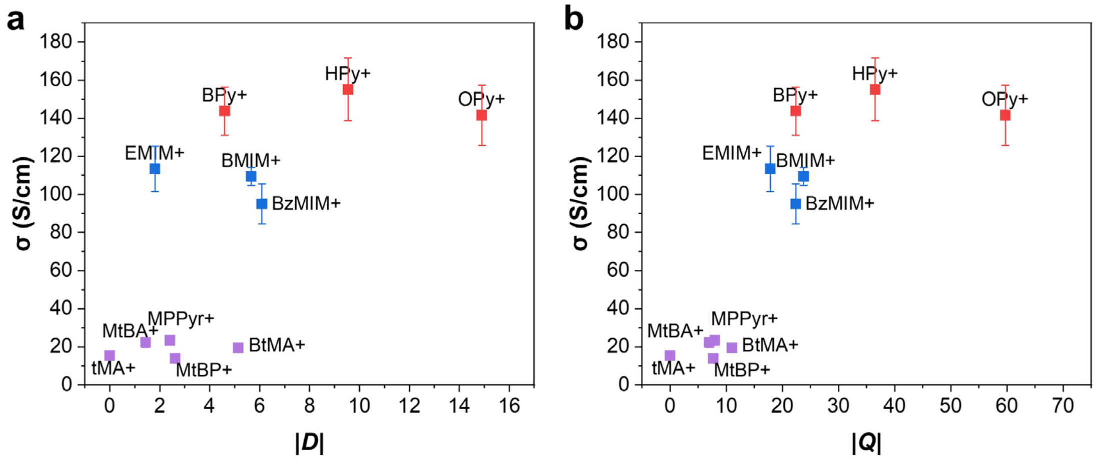

Figure S20. The relationship between a, dipole and b, quadrupole moments with electrical conductivity. Error bars of conductivity indicate the SD of eight experimental replicates.

Section 3. Supplementary Figures and Tables

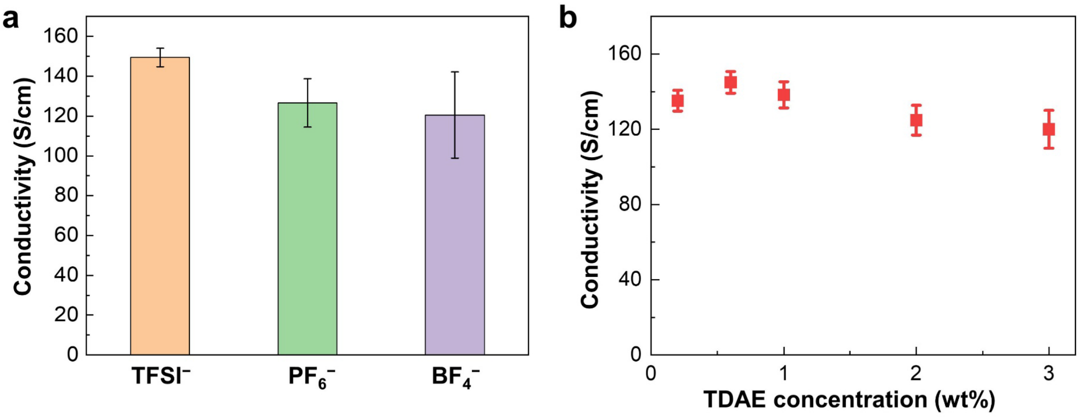

Figure S21. a, Comparison of the electrical conductivities of the ion-exchange doped P(PzDPP-2FT) films with the cation $BMIM^+$ and various anions. When employing $TFSI^-$ salt, the polymer films exhibited higher conductivities. **b**, The conductivity of polymer films doped with TDAE solution at different concentrations. The ion liquid employed in this measurement is $BMIM^+TFSI^-$. At a doping concentration of 0.6 wt% TDAE/$BMIM^+TFSI^-$ solution, the polymer films showed better conductivities. This doping concentration was selected for subsequent characterizations of the ion-exchange doped polymer films. Error bars indicate the SD of eight experimental replicates.

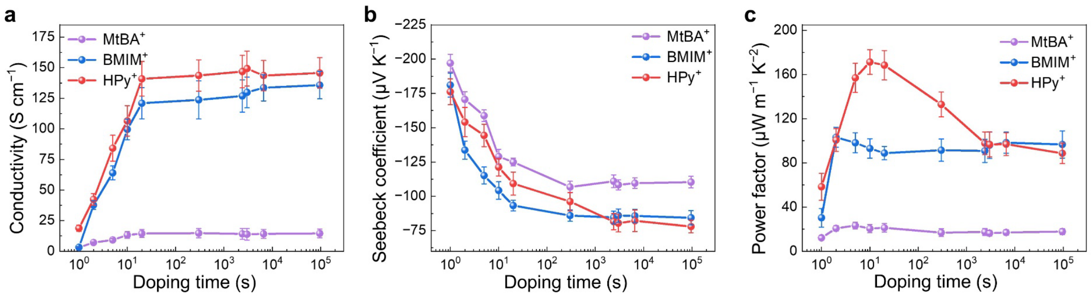

Figure S22. a, Electrical conductivities, **b**, Seebeck coefficients, and **c**, power factors of the P(PzDPP-2FT) films exchanged with three different cations at various doping times. Error bars indicate the SD of eight experimental replicates.

Table S4. Summary of the electrical conductivities and power factors (PFs) of the reported n-doped conjugated polymers.

Polymer	σ_{max} [S cm^{-1}]	PF_{max} [μW m^{-1} K^{-2}]	Reference
**FBDPPV**	14	28	[23]
**UFBDPPV**	22.5	80	[6]
**TBDPPV**	92	76	[24]
**TBDPPV-T**	65	106	[24]
**FBDPPV-OEG**	39.0	36.4	[25]
**TBDOPV-518**	114	236	[26]
**PBFDO**	2000	90	[27]
**LPPV**	1.1	1.96	[28]
**BBL**	8	11	[29]
**P(PzDPP-CT2)**	8.4	57.3	[30]
**P(TDPP-CT2)**	0.39	9.3	[30]
**PDPF**	1.3	4.65	[31]
**PNDTI-BBT-DP**	5.0	14	[32]
**P(gNDI-gT2)**	0.29	0.41	[33]
**TEG-N2200**	0.17	0.40	[34]
**PNDI2TEG-2Tz**	1.8	4.5	[35]
**P(NDI2OD-Tz2)**	0.1	1.5	[36]
**PCNI-BTI**	23.3	10.0	[37]
**PDTzTI**	104	65.7	[38]

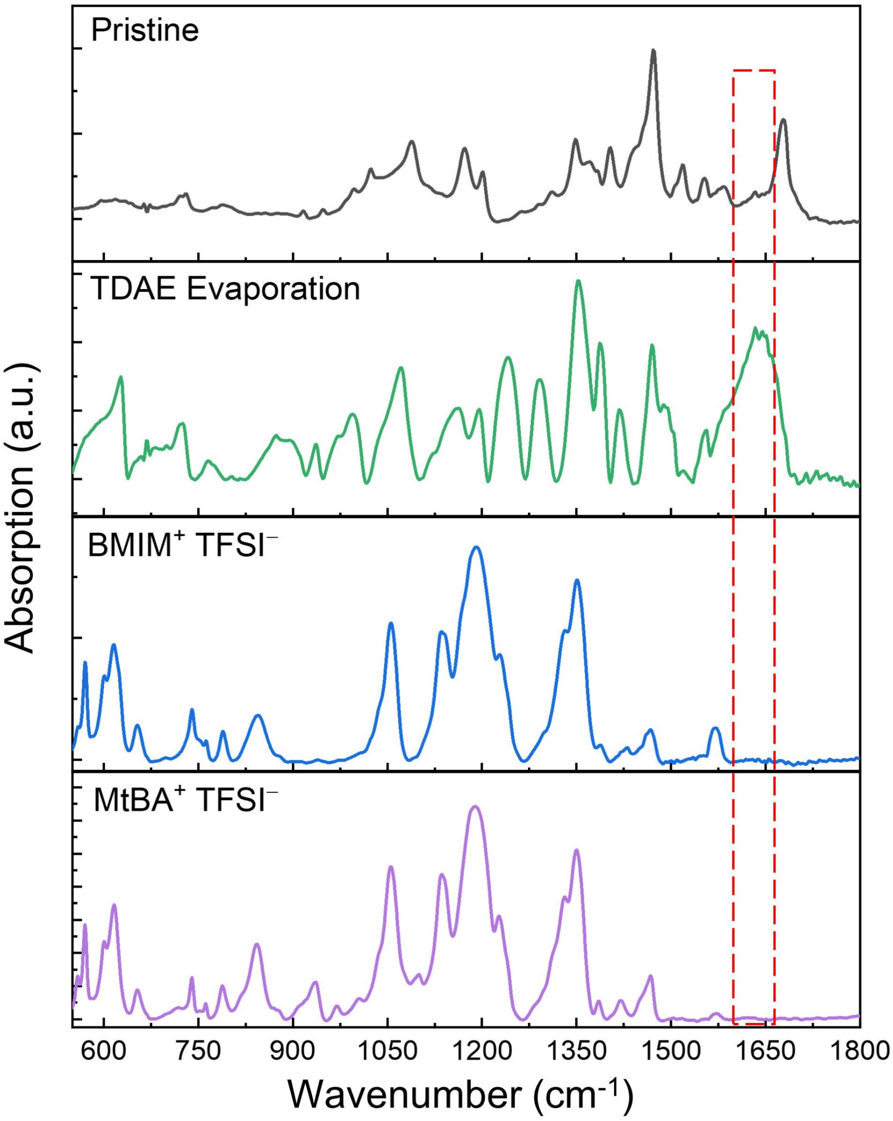

Figure S23. Fourier-transform infrared spectroscopy (FTIR) of the pristine P(PzDPP-2FT), TDAE-doped P(PzDPP-2FT), and P(PzDPP-2FT) exchanged with $BMIM^+$ and $MtBA^+$. After exchanging with cations, the characteristic peaks around 1600-1650 cm^{-1}, corresponding to the TDAE counterion after reacting with polymer, nearly disappeared. This observation suggested that the TDAE dopants were nearly exchanged to the cations of the ion liquid.

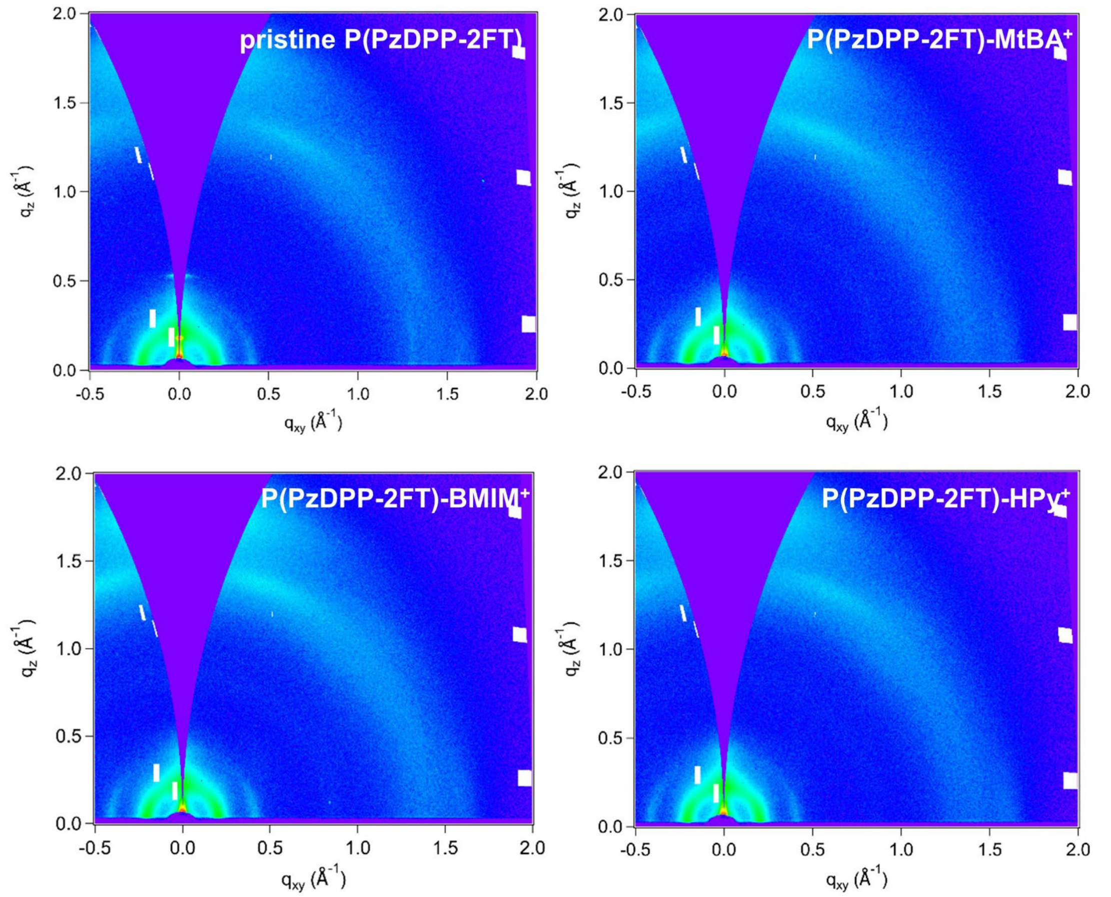

Figure S24. 2D GIWAXS patterns of the pristine P(PzDPP-2FT) and the exchange-doped P(PzDPP-2FT) with $MtBA^+$, $BMIM^+$, and HPy^+ for 5 minutes.

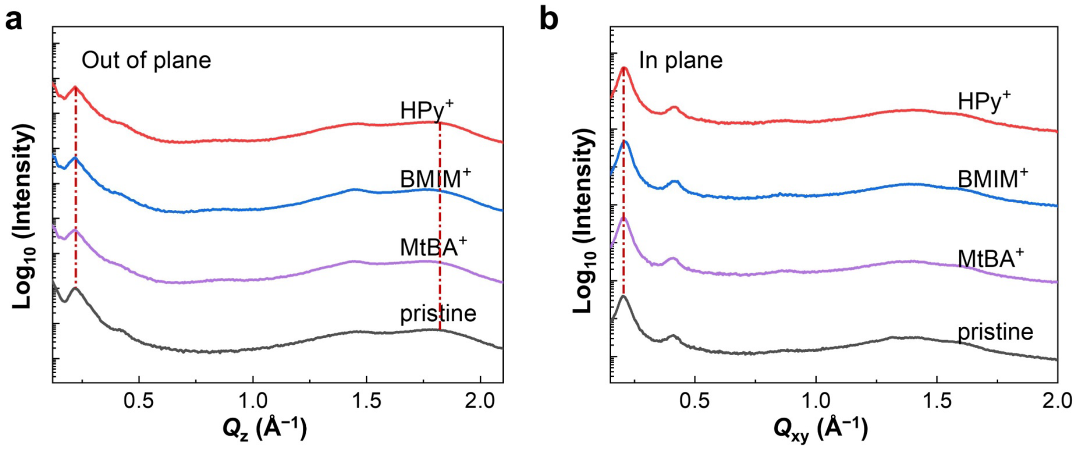

Figure S25. a, out-of-plane and **b,** in-plane 1D GIWAXS plots of the pristine and the exchange-doped P(PzDPP-2FT) with $MtBA^+$, $BMIM^+$, and HPy^+ for 5 minutes.

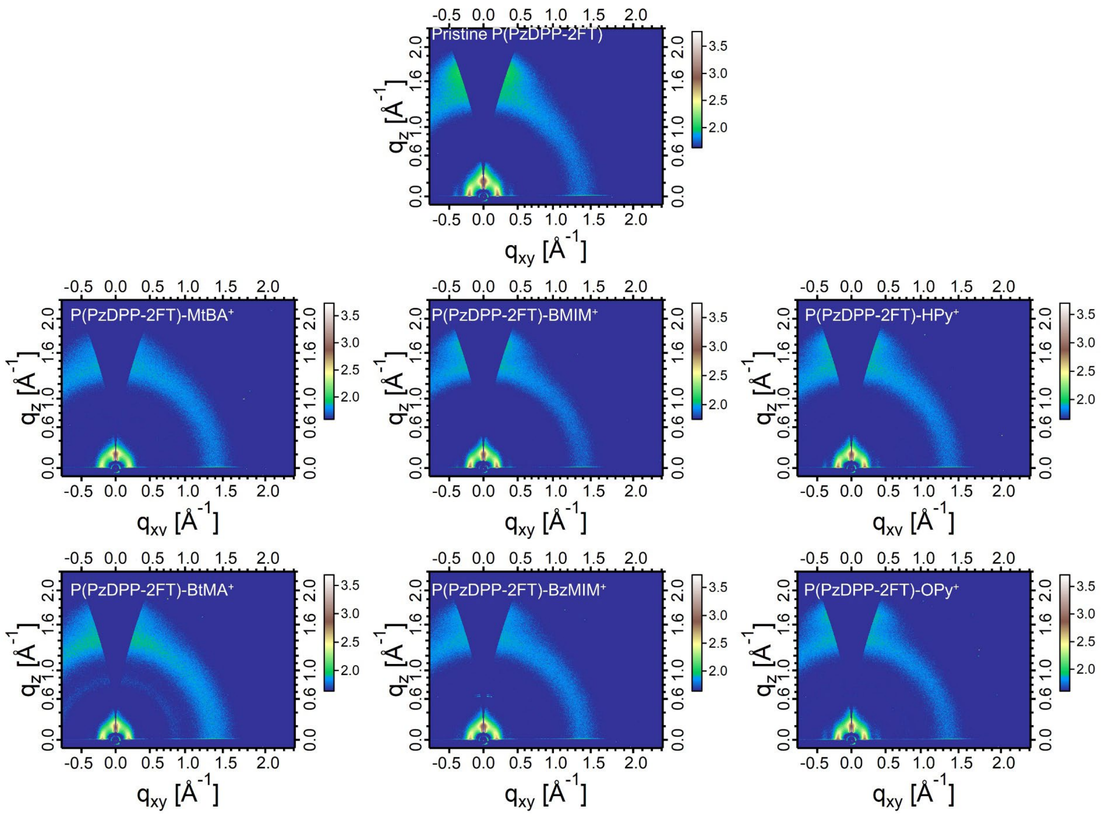

Figure S26. 2D GIWAXS patterns of the pristine P(PzDPP-2FT) and the exchange-doped P(PzDPP-2FT) with various cations for 30 minutes.

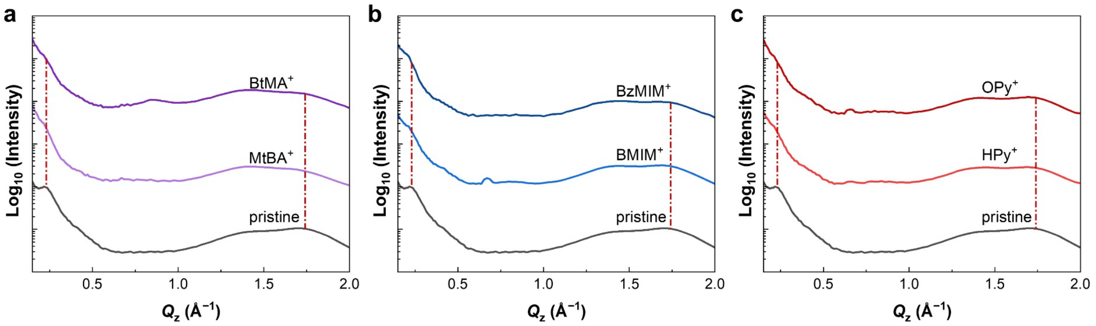

Figure S27. The out-of-plane 1D GIWAXS plots of the pristine P(PzDPP-2FT) and the exchange-doped P(PzDPP-2FT) with various cations for 30 minutes.

Dopants are more likely to initially occupy the amorphous regions due to the difficulty in entering the ordered stacking crystalline region[39]. Previous studies have shown that the π-π/lamellar stacking distances of conjugated polymer remain relatively constant at low doping levels but change significantly at high doping levels[4,40]. This is ascribed to the heterogeneous doping that involves doping of the amorphous domain first before doping of the crystalline domains[41]. In alignment with these findings, we

also observed an expansion in the lamellar stacking distances after exchange-doped with the three counterions ($MtBA^+$, $BMIM^+$, and HPy^+) for 5 minutes, indicating their presence in crystalline regions (Fig. S25). Increasing the doping time to 30 minutes, the lattice expansion further increased (Fig. S27), suggesting that more counterions docked into the crystalline regions. While the π-π distance for the polymer with HPy^+ only slightly increased by 0.02 Å at 30 minutes, the lamellar stacking distance significantly increased by 2.56 Å (Fig. 4c), confirming the docking of HPy^+ into the crystalline region without significantly disrupting the molecular packing.

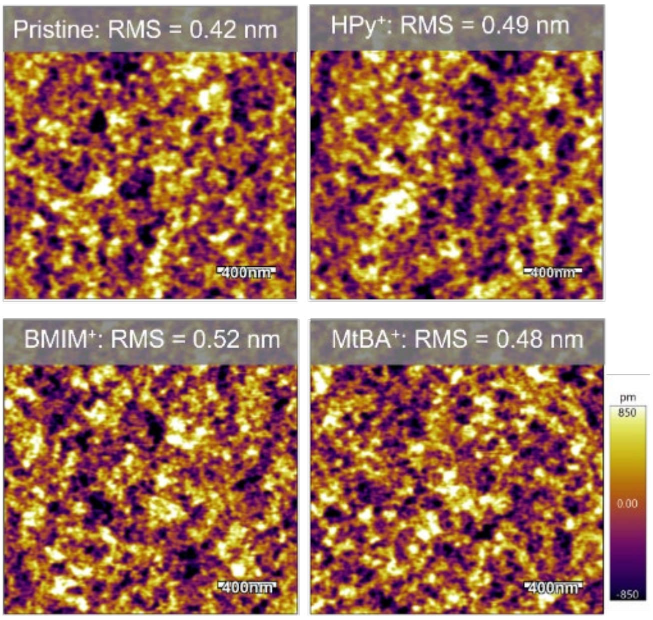

Figure S28. AFM height images of the pristine and the exchange-doped P(PzDPP-2FT) with $MtBA^+$, $BMIM^+$, and HPy^+.

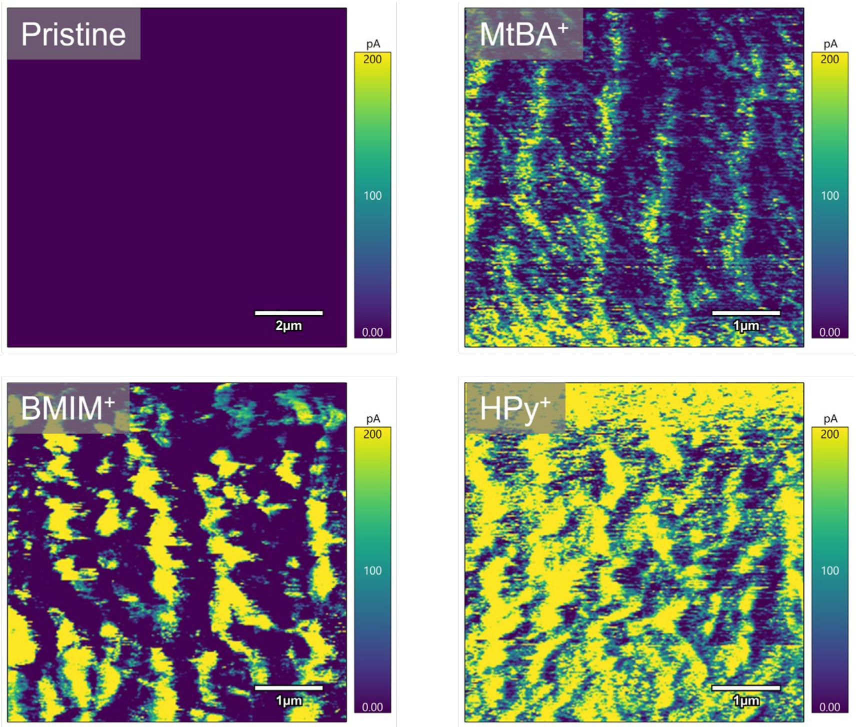

Figure S29. The c-AFM current images of the pristine and the exchange-doped P(PzDPP-2FT) with $MtBA^+$, $BMIM^+$, and HPy^+. The measurement was conducted in a nitrogen atmosphere, and the voltage bias was 100 mV. When exchange-doped with HPy^+, the surface of the polymer film exhibited large connected areas with high currents. In contrast, when exchange-doped with $MtBA^+$, the film showed isolated conducting areas with low currents.

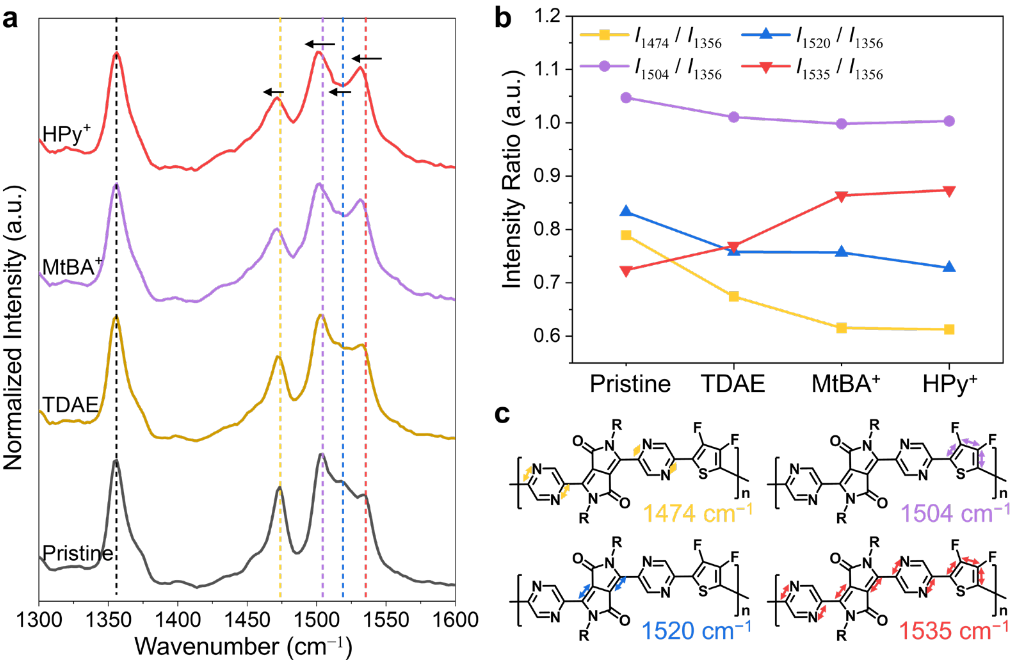

Figure S30. a, Raman spectra of the pristine P(PzDPP-2FT) film, P(PzDPP-2FT) doped with TDAE, and P(PzDPP-2FT) exchange-doped with $MtBA^+$ and HPy^+. All the Raman spectra were excited at 633nm and the Raman intensity was normalized to the peak at 1356 cm^{-1}. b**,** Intensity ratio of different peaks to the peak at 1356 cm^{-1}. **c,** Diagram illustrating the dominant bond stretches associated with the main Raman active vibrational modes of P(PzDPP-2FT) centered around 1474, 1504, 1520, and 1535 cm^{-1}. The peak at 1356 cm^{-1}, associated with the symmetric C-C stretch mode, remained unchanged between the pristine and the doped P(PzDPP-2FT) films. However, all peaks related to the C=C stretch mode (1474, 1504, 1520, and 1535 cm^{-1}) in the polymer with HPy^+ and $MtBA^+$ notably shifted to lower wavenumbers. Additionally, after ion-exchange doping, we observed a decrease in the intensity of the C=C stretching mode (1474, 1504, 1520 cm^{-1}) in the pyrazine, thiophene, and DPP building blocks, while the intensity of the C=C stretching mode (1535 cm^{-1}) across the entire conjugated backbone increased, particularly in the polymer exchanged with HPy^+. These results imply an increase in conjugation length and segmental order of the ion-exchanged polymers[42,43].

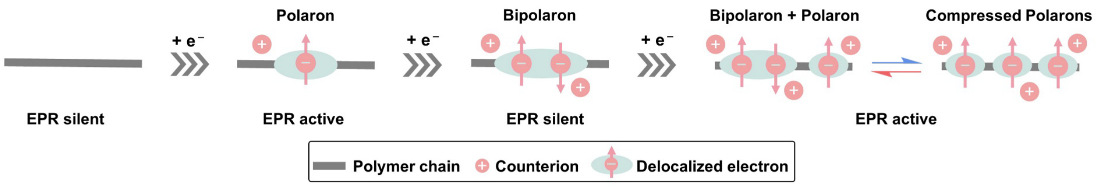

Figure S31. Schematic diagrams illustrate the different possibilities of spin pairing in polymer chains from neutral to different doping states. Upon doping, an additional electron is introduced into one of the polymer chains, leading to the formation of unpaired electrons, known as polarons, which contribute to the EPR signal. Further increasing the doping level, two polarons within the same chain or neighboring chains can couple together to form a bipolaron[44]. Bipolarons are EPR silent and result in a decrease in the spin density (N_S), despite an increase in carrier concentration (N_D)[45]. At higher doping levels, a mixture of bipolarons and newly formed unpaired polarons would give rise to the EPR signal and increase N_S again[46]. However, it is possible that polarons in the same chain may be compressed to shorter lengths to form an array or polaron lattice[47,48]. Although the details of electronic states go beyond the scope of this study, the electronic states of carriers in heavily doped systems have received limited attention. Therefore, we included some of our results on this intriguing topic.

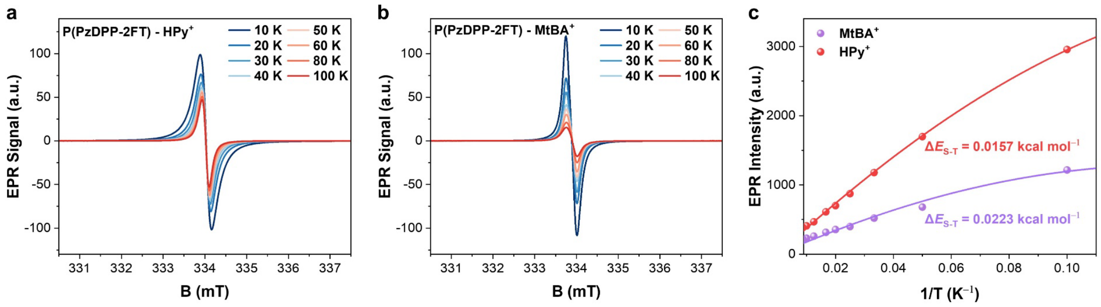

Figure S32. Temperature-dependent EPR of P(PzDPP-2FT) exchanged with **a,** HPy^+ and **b,** $MtBA^+$ and **c,** the EPR integrated intensity and Bleaney-Bowers equation fitting result. A dramatic decrease in EPR integrated intensity with increasing temperature was observed for polymer exchanged with both HPy^+ and $MtBA^+$, suggesting a triplet ground state[49]. We used the Bleaney-Bowers equation to fit their EPR intensity, which provides the energy gap between the singlet and triplet states ($\Delta E_{S\text{-}T}$) of 0.0157 kcal mol^{-1} for the polymer with HPy^+ and 0.0223 kcal mol^{-1} for the polymer with $MtBA^+$. These results suggest that at low temperatures, compressed polarons (existing in triplet state) are more stable than bipolarons (existing in singlet state). As the temperature increases, unpaired compressed polarons gradually combine to form bipolarons. Although distinguishing between compressed polarons and bipolarons becomes more challenging at room temperature, the presence of triplet compressed polarons at low temperatures is evident from these results.

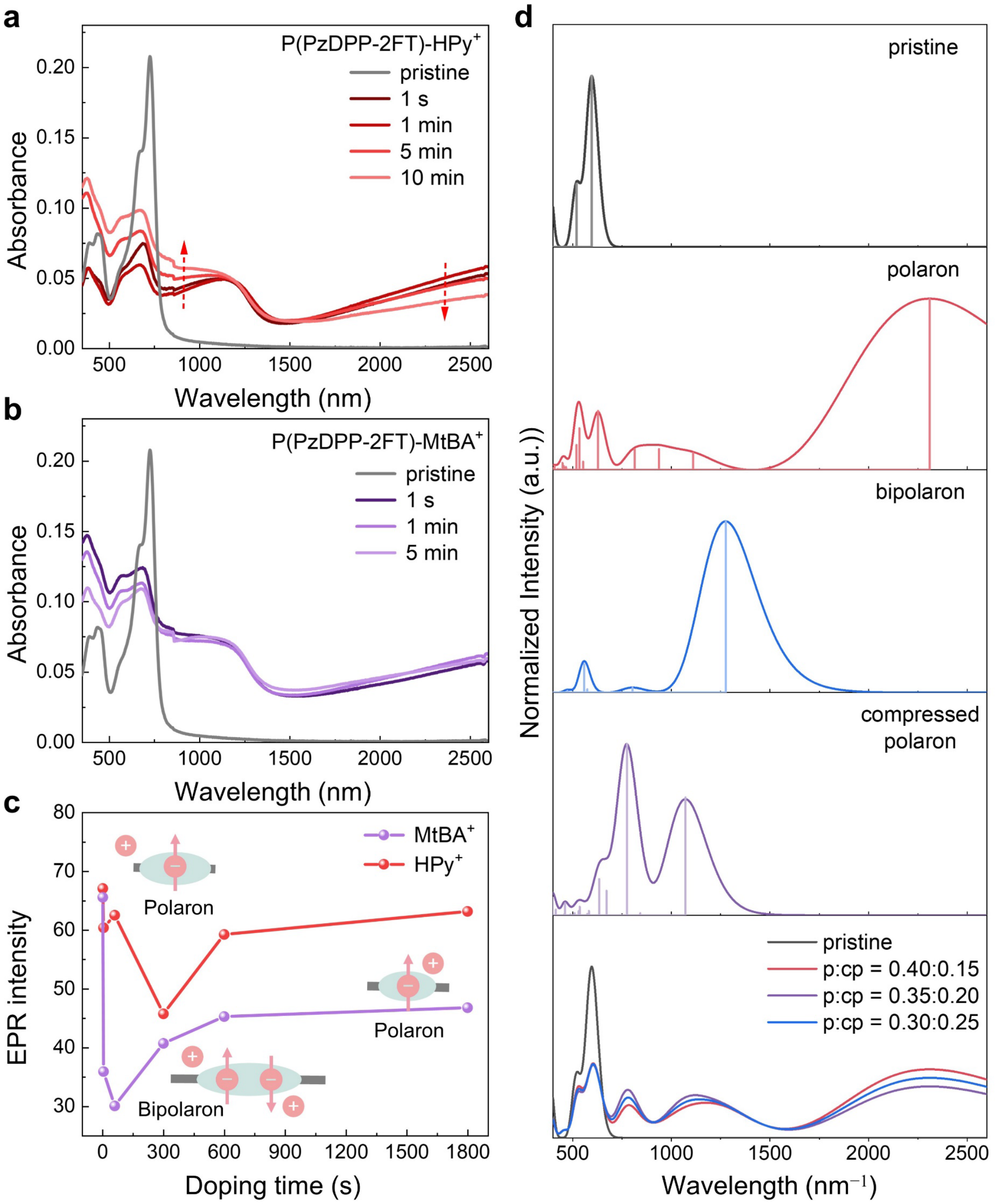

Figure S33. UV-vis-NIR absorption spectra of the polymer films exchange-doped with **a**, $MtBA^+$ and **b**, HPy^+, respectively. **c**, EPR integrated intensity of the polymer films exchange-doped with $MtBA^+$ and HPy^+ at different doping time. **d,** TD-DFT simulated UV-vis-NIR spectra of the P(PzDPP-2FT) trimer at four electronic states: pristine, polaron, bipolaron, and compressed polaron. The bottom figure shows the simulated spectra of the P(PzDPP-2FT) trimer with the four states in different ratios (pristine: polaron: bipolaron: compressed polaron = 0.3: p: 0.15: cp). The TD-DFT calculations were performed at CAM-B3LYP/6-311G(d,p)[50], and the 100 excited states with the lowest energy were calculated for each P(PzDPP-2FT) trimer. Upon doping, two new absorption bands at 2000-2500 nm and 1300 nm were observed (Fig. S33a-b). As the

doping level increased, the film exchanged with HPy^+ showed a gradual decrease in the polaron band, along with the emergence of a new peak at 950 nm (Fig. S33b), indicating the formation of a new type of polaron. The TD-DFT simulated spectra were in good agreement with experimental data throughout and further confirmed the absorption bands of 2000-2500 nm and 1200 nm corresponded to polarons and bipolarons, respectively (Fig. S33c). Additionally, the simulations suggested that the rise in the 900-1200 nm at high doping levels may be attributed to the formation of compressed polarons, which exhibited a blue shift compared to the absorbance of bipolarons. EPR measurements corroborated the changes observed in optical spectra, showing a decrease and then an increase in the spin density (Fig. S33c). At low doping levels, polarons dominated. After increasing doping levels, two adjacent polarons combined to form a spinless bipolaron, leading to a decrease in the EPR integrated intensity. At high doping levels, the formation of compressed polarons leads to an increase in the EPR signal again.

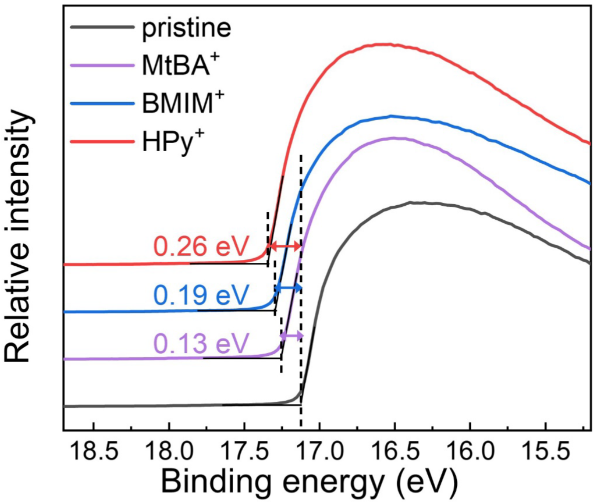

Figure S34. UPS binding energy of the pristine P(PzDPP-2FT) and the exchange-doped P(PzDPP-2FT) with $MtBA^+$, $BMIM^+$, and HPy^+. the P(PzDPP-2FT) exchange-doped with HPy^+ and $BMIM^+$ experienced a larger up-shift in the polymer's Fermi level (0.26 eV for HPy^+ and 0.19 eV for $BMIM^+$, respectively) compared to $MtBA^+$ (0.13 eV).

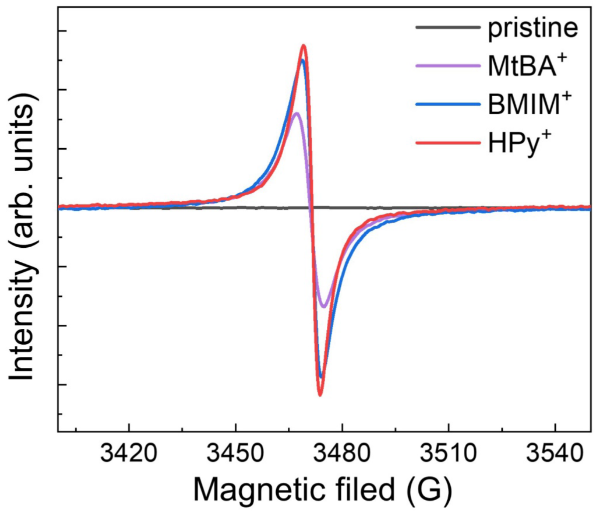

Figure S35. EPR signals of the pristine P(PzDPP-2FT) and the exchange-doped P(PzDPP-2FT) with $MtBA^+$, $BMIM^+$, and HPy^+. The polymer films were immersed in their corresponding doping solutions (0.6 wt% TDAE/ion-liquid) for 5 minutes.

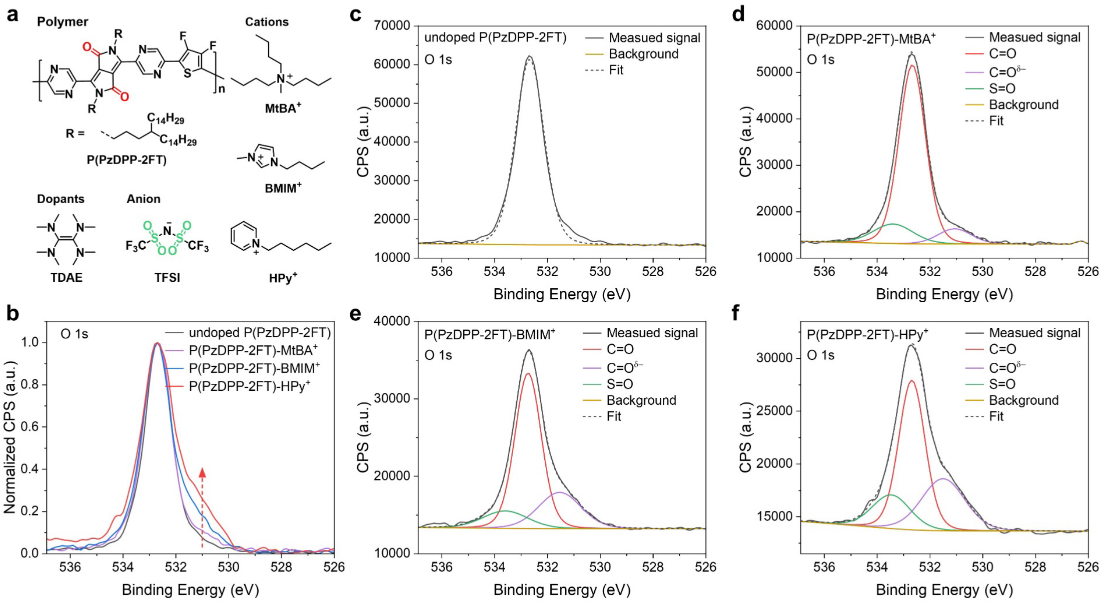

Figure S36. a, Chemical structure of the polymer P(PzDPP-2FT), the dopant TDAE, the three cations ($MtBA^+$, $BMIM^+$, HPy^+), and the corresponding anion $TFSI^-$ in ion liquid. **b**, Normalized O 1*s* XPS spectra for the undoped P(PzDPP-2FT) and the exchange-doped P(PzDPP-2FT) with the three cations. O 1*s* XPS spectra and fitted curves of **c**, the undoped P(PzDPP-2FT) and P(PzDPP-2FT) exchange-doped with **d**, $MtBA^+$, **e**, $BMIM^+$, **f**, HPy^+.

Fig. S36a displays the chemical structures of the materials involved. Fortunately, only the polymer P(PzDPP-2FT) and the anion $TFSI^-$ contain oxygen atoms, allowing for a clear distinction between the C=O in P(PzDPP-2FT) and the S=O in $TFSI^-$. The

O 1*s* peak of S=O in $TFSI^-$ is observed at 533.3 eV [51], while the C=O peak in the polymer is at located 532.6 eV [52]. Therefore, we focused our analysis on the XPS O 1*s* spectra of undoped P(PzDPP-2FT) and the exchange-doped P(PzDPP-2FT) with the three counterions. As shown in Fig. S36c, the undoped P(PzDPP-2FT) exhibited a main peak at 532.6 eV, corresponding to the O in the DPP moiety. After exchange-doping with the three cations, a new peak emerges at 531.5 eV (Fig. S36b), indicating the formation of a new species during the doping process. Since the S=O in $TFSI^-$ exhibits a higher binding energy than the C=O in DPP, this shift to a lower binding energy is attributed to the O atoms on the doped polymer backbone (labeled as $C{=}O^{\delta-}$), which possess fewer positive charges compared to the pristine polymer. Consequently, the charge carrier concentration is determined by the ratio of $C{=}O^{\delta-}$ to the original C=O. Notably, the increase of the $C{=}O^{\delta-}$ peak was more pronounced in the polymer exchange-doped with HPy^+ compared to that with $MtBA^+$ (Fig. S36b), indicating a higher doping level. This observation is corroborated by the fitting results, where the ratio of $C{=}O^{\delta-}/C{=}O$ in the polymer exchange-doped with HPy^+ was 0.571, significantly higher than the 0.108 ratio in the polymer exchange-doped with $MtBA^+$. Therefore, the estimated carrier concentration in the polymer exchange-doped with HPy^+ is 1.64×10^{20} cm^{-3}, which is 3.7 times higher than that in the polymer exchange-doped with $MtBA^+$ (as detailed in Table S5).

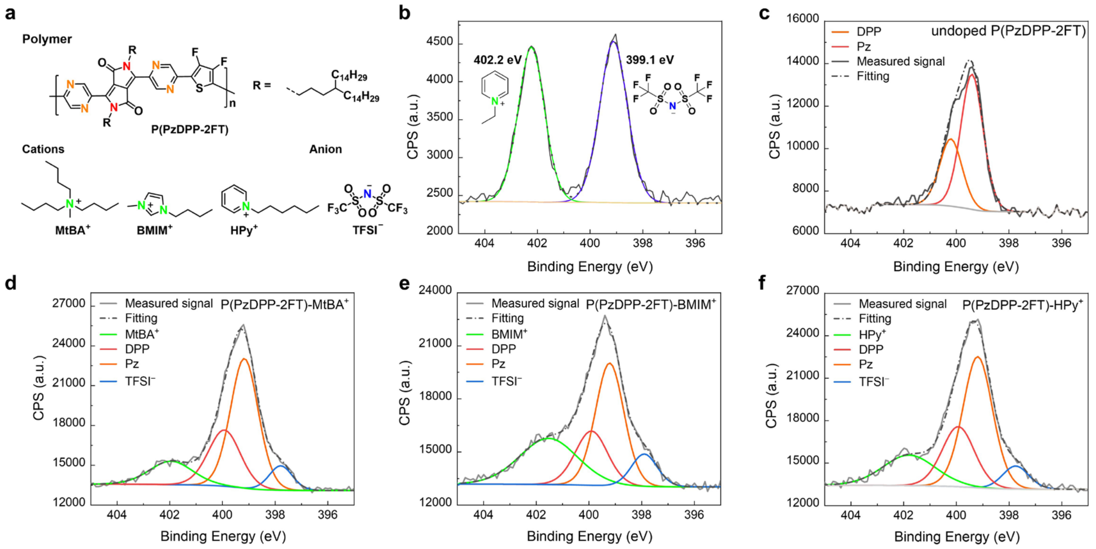

Figure S37. a, Chemical structure of the polymer P(PzDPP-2FT), the dopant TDAE, the three cations ($MtBA^+$, $BMIM^+$, HPy^+), and the anion $TFSI^-$. **b,** N(1s) XPS spectra and fitted curves of **c**, the undoped P(PzDPP-2FT) and P(PzDPP-2FT) exchange-doped with **d**, $MtBA^+$, **e**, $BMIM^+$, **f**, HPy^+.

To calculate the counterion density, we have utilized XPS to analyze and characterize the atomic species of counterions. We first excluded the interference of the residual TDAE dopants. Our Fourier-transform infrared spectroscopy (FTIR) analysis indicated a significant reduction in the characteristic peaks of TDAE (1600-1650 cm^{-1}) after ion-exchanging (Fig. S23), suggesting a near-complete exchange of $TDAE^-$ by cations from the ion liquids. After ion-exchange doping, four distinct nitrogen (N) environments were observed in the polymer films, as depicted in Fig. S37a: N from the DPP moiety, N from the pyrazine, N^+ from cations, and N^- from $TFSI^-$. The N(1*s*) XPS data for the pure ion liquid EPy^+-$TFSI^-$ revealed distinct N^+ and N^- peaks at 402.2 eV and 399.1 eV, respectively (Fig. S37b). In contrast, the neutral N from the polymer backbone exhibited double peaks at 400.2 eV and 399.4 eV (Fig. S37c), allowing for a clear distinction between the neutral N from the polymer and N^+/ N^- from ions. Fig. S37d-f illustrates the XPS N (1s) spectra of P(PzDPP-2FT) exchange-doped with the three counterions. The emergence of two new peaks at approximately 402 eV and 398 eV corresponds to N^+ of the cations and N^- from $TFSI^-$, respectively. The slight shift of 0.2 eV in the neutral N from the undoped polymer to lower binding energy after n-

doping indicates the formation of new negatively charged N species during the doping process. This shift, however, is minimal compared to the peaks associated with cations and anions and does not impact the cation/polymer ratio. For simplicity, we make no distinctions between the neutral N and charged N species of the polymer and take them as a unity. Based on the above analysis, the counterion concentration $n_{counterion}$ within the polymer can be estimated using the following formula:

$$n_{counterion} = \frac{S_{cation} - S_{TFSI^-}}{S_{poly}} n_{poly}$$

Here, n_{poly} is the number of N atoms per polymer monomer, while S_{cation}, S_{TFSI^-}, and S_{poly} correspond to the signals from cations, anion $TFSI^-$, and the polymer, respectively. It is important to note that the cation concentration calculation for $BMIM^+$ should account for its two N atoms per cation, thus dividing S_{cation} by 2. The estimated counterion concentrations are summarized in Table S5. The polymer exchange-doped with HPy^+ exhibits an estimated counterion concentration of 7.66×10^{19} cm^{-3}, which is twice that of the polymer exchange-doped with $MtBA^+$ (3.70×10^{19} cm^{-3}). As a result, the estimated average counterion-counterion distance ($R_{\mathrm{counterion}} = n_{\mathrm{counterion}}^{-1/3}$) of HPy^+ is 23.5 Å, while $MtBA^+$ exhibits a larger distance of 30.0 Å.

Table S5. Charge carrier densities measured by AC-Hall, EPR, and XPS. And the corresponding estimated average ion-ion distance ($R = n^{-1/3}$).

	n_{EPR} (cm^{-3})	$n_{\mathrm{AC\text{-}Hall}}$ (cm^{-3})	n_{XPS} (cm^{-3})	$n_{\mathrm{XPS\text{-}ion}}$ (cm^{-3})	$R_{\mathrm{AC\text{-}Hall}}$ (Å)	R_{XPS} (Å)	$R_{\mathrm{XPS\text{-}ion}}$ (Å)
$MtBA^+$	1.14×10^{19}	9.13×10^{19}	4.40×10^{19}	3.70×10^{19}	22.2	28.3	30.0
$BMIM^+$	2.04×10^{19}	6.20×10^{20}	1.23×10^{20}	4.18×10^{19}	11.7	20.1	28.8
HPy^+	1.49×10^{19}	9.37×10^{20}	1.64×10^{20}	7.66×10^{19}	10.2	18.3	23.5

Quantitatively defining charge carrier concentration in these materials is indeed challenging due to the limitations and specific characteristics of the commonly used measurement techniques. To measure the charge carrier concentration of the doped polymer, we used three different methods: EPR spectroscopy, AC-Hall test, and XPS. EPR was used to evaluate the number of unpaired electrons in the polymer. We observed that the polymer exchange with $BMIM^+$ and HPy^+ showed stronger signals than that exchanged with MtBA (Fig. S35). The double-integrated intensity of the experimental

spectrum was used to measure the number of spins with TEMPO as the reference standard[53]. The calculated spin density of $BMIM^+$ and HPy^+ was slightly higher than that exchanged with $MtBA^+$. However, EPR spectra are effective in measuring unpaired electrons, such as polarons, yet at high doping levels, species like bipolarons, which are EPR silent, remain undetectable. AC-Hall measurements provided further insight into the charge concentration. We found that the charge carrier concentration of the films exchanged doping with $BMIM^+$ and HPy^+ was about an order of magnitude higher than that with $MtBA^+$ (9.13×10^{19} cm^{-3}), with values of about 6.02×10^{20} cm^{-3} and 9.37×10^{20} cm^{-3}, respectively. However, the AC-Hall test typically overestimated the carrier density due to partial screening by hopping carriers[54]. Therefore, we used XPS to characterize atomic species and calculate the charge carrier concentration based on the ratio of peak integrals, proportional to the molar ratio of atoms within each species. After exchange doping, the transition ratio of the intrinsic polymer can be obtained by dividing the peaks (Fig. S36). The charge carrier density of the films exchanged doping with $BMIM^+$, HPy^+, and $MtBA^+$ were 1.48×10^{20} cm^{-3}, 1.55×10^{20} cm^{-3} and 5.25×10^{19} cm^{-3}, respectively. This suggests the delocalization of one electron over 3-4 polymer repeat units. Our results suggest that the high conductivities of the films exchanged with $BMIM^+$ and HPy^+ were due to the high mobility and high charge carrier concentration.

Table S6. Comparison of the electrical performance of the polymer exchange-doped with $MtBA^+$ and HPy^+. Error bars indicate the SD of eight experimental replicates.

Measurements	$MtBA^+$	HPy^+	Factors ($HPy^+/MtBA^+$)
σ	23.4 ± 2.4	155.2 ± 16.5	5.4~8.2
($S\ cm^{-1}$)	14.8 ± 3.8	149.2 ± 14.3	7.3~14.9
μ_{Hall} ($cm^2\ V^{-1}\ s^{-1}$)	0.21 ± 0.05	0.53 ± 0.14	1.5~4.2
n_{Hall} (cm^{-3})	9.12×10^{19}	9.37×10^{20}	10.2
n_{XPS} (cm^{-3})	4.40×10^{19}	1.64×10^{20}	3.7
n_{EPR} (cm^{-3})	1.14×10^{19}	1.49×10^{19}	1.3

Electrical conductivity (σ) is a function of both carrier concentration (n) and mobility (μ), as described by the equation $\sigma = en\mu$. Our data indicates that the polymer exchange-doped with HPy^+ exhibited a 1.5 to 4.2 fold increase in μ and a 1.3 to 10.2 fold increase in n compared to $MtBA^+$. EPR measurements tend to underestimate the charge carrier concentration, while Hall measurements generally overestimate it. Taking into account the relatively accurate results from XPS analysis, where the n of HPy^+ is 3.7 times that of $MtBA^+$, and multiplying it by the 1.5 to 4.2 fold increase in mobility, we expect the conductivity to be higher by a factor of 5.5 to 15.5. This expectation aligns well with our measurement results, showing a 5.4 to 14.9 fold increase in conductivity.

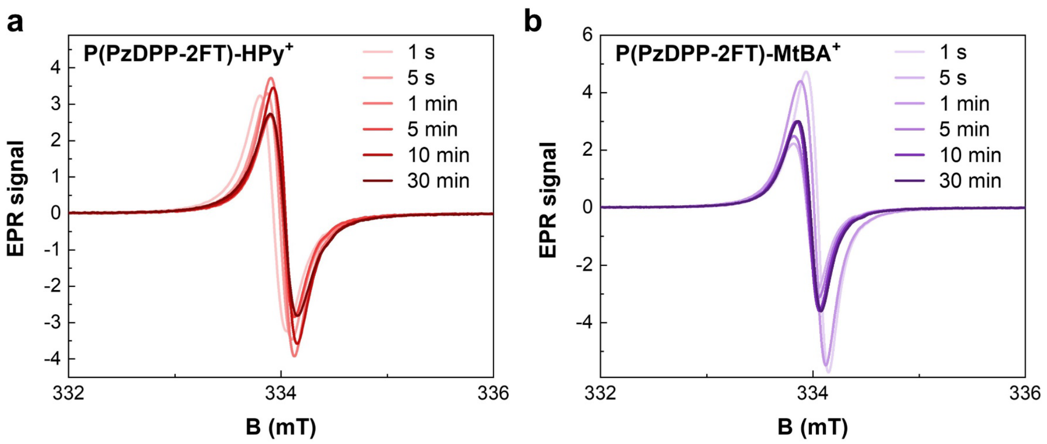

Figure S38. EPR measurements of P(PzDPP-2FT) exchange-doped by $MtBA^+$ and HPy^+ with different doping times.

Table S7. The fitted transport parameters for P(PzDPP-2FT) exchange doped with $MtBA^+$, $BMIM^+$, and HPy^+ using the SLoT model.

	$MtBA^+$	$BMIM^+$	HPy^+
σ_0 (S/cm)	7.50	44.0	60.0
W_H^{max} (meV)		130	
W_H^{slope} (meV)	151	180	195
c_d	0.630	0.369	0.284
c_{XPS}	0.117	0.328	0.344
$W_H(c_{XPS})$ (meV)	55.5	5.60	0.00

σ_0 is the function of the material's charge carrier mobility and effective mass[55,56], which shifts the entire S-σ curve. W_H^{max} and W_H^{slope} are the maximum localization energy and the rate at which localization decays with increasing carrier concentration, respectively. c_d is the carrier ratio where the delocalized transport is achieved. $W_H(c_{XPS})$ is the calculated potential energy well at the XPS measured charge carrier ratio c_{XPS}. For comparison, we assumed that the W_H^{max} of the same polymer P(PzDPP-2FT) should be consistent when exchange-doped with different ion liquids. We observed that when P(PzDPP-2FT) was exchanged with HPy^+, it exhibited higher mobility (indicated by a larger σ_0) and facilitated easier achievement of delocalized transport with larger W_H^{slope} and lower c_d compared to the other two cations. Eventually, using the XPS-measured carrier ratio, the W_H remained at 55 meV for P(PzDPP-2FT) exchanged with $MtBA^+$ whereas in P(PzDPP-2FT)-HPy^+, it approached zero.

References

1. Frisch, M.J. et al. Gaussian 16, Revision, C.01, Gaussian, Inc., Wallingford CT, 2016.
2. Wu, G., Robertson, D.H., Brooks Iii, C.L., Vieth, M. Detailed analysis of grid-based molecular docking: A case study of cdocker—a charmm-based md docking algorithm. *J. Comput. Chem.* **24**, 1549-1562 (2003).
3. Yan, X. et al. Approaching disorder-tolerant semiconducting polymers. *Nat. Commun.* **12**, 5723 (2021).
4. Yang, C.-Y. et al. A thermally activated and highly miscible dopant for n-type organic thermoelectrics. *Nat. Commun.* **11**, 3292 (2020).
5. Venkateshvaran, D. et al. Approaching disorder-free transport in high-mobility conjugated polymers. *Nature* **515**, 384-388 (2014).
6. Lu, Y. et al. The critical role of dopant cations in electrical conductivity and thermoelectric performance of n-doped polymers. *J. Am. Chem. Soc.* **142**, 15340-15348 (2020).
7. Mukasa, D. et al. A computationally assisted approach for designing wearable biosensors toward non-invasive personalized molecular analysis. *Adv. Mater.*, 2212161 (2023).
8. Materials studio v6.0.0 Accelrys Software Inc., San Diego, CA, 2011.
9. Fratini, S., Nikolka, M., Salleo, A., Schweicher, G., Sirringhaus, H. Charge transport in high-mobility conjugated polymers and molecular semiconductors. *Nat. Mater.* **19**, 491-502 (2020).
10. Honig, B., Nicholls, A. Classical electrostatics in biology and chemistry. *Science* **268**, 1144-1149 (1995).
11. Halgren, T.A. The representation of van der Waals (vdW) interactions in molecular mechanics force fields: Potential form, combination rules, and vdW parameters. *J. Am. Chem. Soc.* **114**, 7827-7843 (1992).
12. Lu, T., Chen, Q. Simple, efficient, and universal energy decomposition analysis method based on dispersion-corrected density functional theory. *J. Phys. Chem. A* **127**, 7023-7035 (2023).
13. Li, P., Shi, J., Lei, Y., Huang, Z., Lei, T. Switching p-type to high-performance n-type organic electrochemical transistors via doped state engineering. *Nat. Commun.* **13**, 5970 (2022).
14. Jackson, N.E., Savoie, B.M., Kohlstedt, K.L., Olvera de la Cruz, M., Schatz, G.C., Chen, L.X., Ratner, M.A. Controlling conformations of conjugated polymers and small molecules: The role of nonbonding interactions. *J. Am. Chem. Soc.* **135**, 10475-10483 (2013).
15. Huang, H., Yang, L., Facchetti, A., Marks, T.J. Organic and polymeric semiconductors enhanced by noncovalent conformational locks. *Chem. Rev.* **117**, 10291-10318 (2017).
16. Lefebvre, C., Rubez, G., Khartabil, H., Boisson, J.-C., Contreras-García, J., Hénon, E. Accurately extracting the signature of intermolecular interactions present in the NCI plot of the reduced density gradient versus electron density. *Phys. Chem. Chem. Phys.* **19**, 17928-17936 (2017).
17. Jiang, L., Hirst, J.D., Do, H. Structure–property relationships in amorphous thieno[3,2-b]thiophene–diketopyrrolopyrrole–thiophene-containing polymers. *J. Phys. Chem. C*

126, 10842-10854 (2022).

18. Qin, T., Troisi, A. Relation between structure and electronic properties of amorphous MEH-PPV polymers. *J. Am. Chem. Soc.* **135**, 11247-11256 (2013).
19. Nikolka, M. et al. High-mobility, trap-free charge transport in conjugated polymer diodes. *Nat. Commun.* **10**, 2122 (2019).
20. Lemaur, V., Cornil, J., Lazzaroni, R., Sirringhaus, H., Beljonne, D., Olivier, Y. Resilience to conformational fluctuations controls energetic disorder in conjugated polymer materials: Insights from atomistic simulations. *Chem. Mater.* **31**, 6889-6899 (2019).
21. Armleder, J., Neumann, T., Symalla, F., Strunk, T., Olivares Peña, J.E., Wenzel, W., Fediai, A. Controlling doping efficiency in organic semiconductors by tuning short-range overscreening. *Nat. Commun.* **14**, 1356 (2023).
22. Schwarze, M. et al. Molecular parameters responsible for thermally activated transport in doped organic semiconductors. *Nat. Mater.* **18**, 242-248 (2019).
23. Shi, K. et al. Toward high performance n-type thermoelectric materials by rational modification of BDPPV backbones. *J. Am. Chem. Soc.* **137**, 6979-6982 (2015).
24. Lu, Y. et al. Persistent conjugated backbone and disordered lamellar packing impart polymers with efficient n-doping and high conductivities. *Adv. Mater.* **33**, 2005946 (2021).
25. Wang, X.-Y. et al. Density of states engineering of n-doped conjugated polymers for high charge transport performances. *Adv. Mater.* **35**, 2300634 (2023).
26. Yu, Z.-D. et al. High n-type and p-type conductivities and power factors achieved in a single conjugated polymer. *Sci. Adv.* **9**, eadf3495 (2023).
27. Tang, H. et al. A solution-processed n-type conducting polymer with ultrahigh conductivity. *Nature* **611**, 271-277 (2022).
28. Lu, Y. et al. Rigid coplanar polymers for stable n-type polymer thermoelectrics. *Angew. Chem. Int. Ed.* **58**, 11390-11394 (2019).
29. Yang, C.-Y. et al. A high-conductivity n-type polymeric ink for printed electronics. *Nat. Commun.* **12**, 2354 (2021).
30. Yan, X. et al. Pyrazine-flanked diketopyrrolopyrrole (DPP): A new polymer building block for high-performance n-type organic thermoelectrics. *J. Am. Chem. Soc.* **141**, 20215-20221 (2019).
31. Yang, C.-Y. et al. Enhancing the n-type conductivity and thermoelectric performance of donor–acceptor copolymers through donor engineering. *Adv. Mater.* **30**, 1802850 (2018).
32. Wang, Y., Nakano, M., Michinobu, T., Kiyota, Y., Mori, T., Takimiya, K. Naphthodithiophenediimide–benzobisthiadiazole-based polymers: Versatile n-type materials for field-effect transistors and thermoelectric devices. *Macromolecules* **50**, 857-864 (2017).
33. Kiefer, D. et al. Enhanced n-doping efficiency of a naphthalenediimide-based copolymer through polar side chains for organic thermoelectrics. *ACS Energy Lett.* **3**, 278-285 (2018).
34. Liu, J. et al. Enhancing molecular n-type doping of donor–acceptor copolymers by tailoring side chains. *Adv. Mater.* **30**, 1704630 (2018).

35. Liu, J. et al. N-type organic thermoelectrics of donor–acceptor copolymers: Improved power factor by molecular tailoring of the density of states. *Adv. Mater.* **30**, 1804290 (2018).
36. Wang, S. et al. A chemically doped naphthalenediimide-bithiazole polymer for n-type organic thermoelectrics. *Adv. Mater.* **30**, 1801898 (2018).
37. Feng, K. et al. Cyano-functionalized bithiophene imide-based n-type polymer semiconductors: Synthesis, structure–property correlations, and thermoelectric performance. *J. Am. Chem. Soc.* **143**, 1539-1552 (2021).
38. Guo, H. et al. Transition metal-catalysed molecular n-doping of organic semiconductors. *Nature* **599**, 67-73 (2021).
39. Zhong, Y. et al. Preferential location of dopants in the amorphous phase of oriented regioregular poly(3-hexylthiophene-2,5-diyl) films helps reach charge conductivities of 3000 S cm^{-1}. *Adv. Funct. Mater.* **32**, 2202075 (2022).
40. Jacobs, I.E. et al. High-efficiency ion-exchange doping of conducting polymers. *Adv. Mater.* **34**, 2102988 (2022).
41. Chen, C. et al. Observation of weak counterion size dependence of thermoelectric transport in ion exchange doped conducting polymers across a wide range of conductivities. *Adv. Energy Mater.* **13**, 2202797 (2023).
42. Wood, S., Hollis, J.R., Kim, J.-S. Raman spectroscopy as an advanced structural nanoprobe for conjugated molecular semiconductors. *J. Phys. D: Appl. Phys.* **50**, 073001 (2017).
43. Yu, S.H., Park, K.H., Kim, Y.-H., Chung, D.S., Kwon, S.-K. Fine molecular tuning of diketopyrrolopyrrole-based polymer semiconductors for efficient charge transport: Effects of intramolecular conjugation structure. *Macromolecules* **50**, 4227-4234 (2017).
44. Swager, T.M. 50th anniversary perspective: Conducting/semiconducting conjugated polymers. A personal perspective on the past and the future. *Macromolecules* **50**, 4867-4886 (2017).
45. Miller, L.L., Mann, K.R. π-dimers and π-stacks in solution and in conducting polymers. *Acc. Chem. Res.* **29**, 417-423 (1996).
46. Furukawa, Y. Electronic absorption and vibrational spectroscopies of conjugated conducting polymers. *J. Phys. Chem.* **100**, 15644-15653 (1996).
47. Kivelson, S., Heeger, A.J. First-order transition to a metallic state in polyacetylene: A strong-coupling polaronic metal. *Phys. Rev. Lett.* **55**, 308-311 (1985).
48. Bakalis, J., Cook, A.R., Asaoka, S., Forster, M., Scherf, U., Miller, J.R. Polarons, compressed polarons, and bipolarons in conjugated polymers. *J. Phys. Chem. C* **118**, 114-125 (2014).
49. Abe, M. Diradicals. *Chem. Rev.* **113**, 7011-7088 (2013).
50. Crawford, A.G. et al. Experimental and theoretical studies of the photophysical properties of 2- and 2,7-functionalized pyrene derivatives. *J. Am. Chem. Soc.* **133**, 13349-13362 (2011).
51. Forster-Tonigold, K., Buchner, F., Bansmann, J., Behm, R.J., Groß, A. A combined XPS and computational study of the chemical reduction of BMP-TFSI by lithium. *Batteries & Supercaps* **5**, e202200307 (2022).
52. Yue, H. et al. Synthesis and characterization of novel d-a type neutral blue

electrochromic polymers containing pyrrole[3-c]pyrrole-1,4-diketone as the acceptor units and the aromatics donor units with different planar structures. *Polymers* **11**, 2023 (2019).

53. Chen, X.-X. et al. High-mobility semiconducting polymers with different spin ground states. *Nat. Commun.* **13**, 2258 (2022).
54. Yi, H.T., Gartstein, Y.N., Podzorov, V. Charge carrier coherence and hall effect in organic semiconductors. *Sci. Rep.* **6**, 23650 (2016).
55. Gregory, S.A. et al. Quantifying charge carrier localization in chemically doped semiconducting polymers. *Nat. Mater.* **20**, 1414-1421 (2021).
56. Kang, S.D., Snyder, G.J. Charge-transport model for conducting polymers. *Nat. Mater.* **16**, 252-257 (2017).
